# What we learned from the Dust Bowl: lessons in science, policy, and adaptation

**DOI:** 10.1007/s11111-013-0190-z

**Published:** 2013-08-28

**Authors:** Robert A. McLeman, Juliette Dupre, Lea Berrang Ford, James Ford, Konrad Gajewski, Gregory Marchildon

**Affiliations:** 1 Department of Geography and Environmental Studies, Wilfrid Laurier University, 75 University Avenue West, Waterloo, ON N2L 3C5 Canada; 2 Department of Geography, Burnside Hall, McGill University, 805 Sherbrooke Street West, Montreal, QC H3A 0B9 Canada; 3 Department of Geography, University of Ottawa, Simard Hall Room 047, 60 University, Ottawa, ON K1N 6N5 Canada; 4 Canada Research Chair in Public Policy and Economic History, Johnson-Shoyama School of Public Policy, University of Regina, 110-2 Research Drive, Regina, SK S4S 7H1 Canada

**Keywords:** Climate adaptation, Dirty Thirties, Drought, Dust Bowl, Great Plains, Great Depression

## Abstract

This article provides a review and synthesis of scholarly knowledge of Depression-era droughts on the North American Great Plains, a time and place known colloquially as the Dust Bowl era or the Dirty Thirties. Recent events, including the 2008 financial crisis, severe droughts in the US corn belt, and the release of a popular documentary film, have spawned a resurgence in public interest in the Dust Bowl. Events of the Dust Bowl era have also proven in recent years to be of considerable interest to scholars researching phenomena related to global environmental change, including atmospheric circulation, drought modeling, land management, institutional behavior, adaptation processes, and human migration. In this review, we draw out common themes in terms of not only what natural and social scientists have learned about the Dust Bowl era itself, but also how insights gained from the study of that period are helping to enhance our understanding of climate–human relations more generally.

**Electronic supplementary material:**

The online version of this article (doi:10.1007/s11111-013-0190-z) contains supplementary material, which is available to authorized users.

## Introduction

During the worst years of the Great Depression, large areas of the North American Great Plains experienced severe, multi-year droughts that led to soil erosion, dust storms, farm abandonments, personal hardships, and distress migration on scales not previously seen. Known colloquially as the “Dirty Thirties” or “the Dust Bowl years,” they captured an important place in wider popular memory through John Steinbeck’s *The Grapes of Wrath* (1939) and the iconic images of US Farm Security Administration photographers. The subject of hundreds of popular books and films in subsequent decades, “the worst hard time” as author Timothy Egan (2006) has called it, has enjoyed a resurgence in public attention following the 2008 financial crisis, recent droughts in the US corn belt, and the November 2012 release of the Ken Burns documentary film *The Dust Bowl*. What is also notable, and is the focus of the present article, is that there has been considerable growth in scholarship on the subject in recent years, across a wide range of natural and social science disciplines. This includes one subset of works that seeks to explain or interpret the causes and consequences of events of the 1930s and another that uses events of the Dust Bowl era as learning vehicles and analogs to test datasets, methods, and theories with broader applicability to global change research. There are a number of potential explanations for the increase in scholarly attention. These include, but are not limited to, growing interest in the causes of droughts and their return frequency, the availability of new atmospheric datasets, greater analog-based research on the human dimensions of climate change, new directions in environmental migration research, and the growth in global environmental change scholarship more generally. Comparisons of the 2008 financial crisis to the Great Depression and the effects of recent droughts on global food prices are additional elements that influence current Dust Bowl research.

In this article, we review and synthesize the current state of scholarly knowledge of Dust Bowl era droughts, their ecological or socio-economic impacts, and the use of events from that period as a means to develop insights into related phenomena. We have sought to draw out common themes in terms of not only what natural and social scientists have learned about the Dust Bowl era itself, but also how insights gained from the study of that period are helping to enhance our understanding of climate–human relations more generally. We have also sought to identify potential avenues for future research, considering in particular future policymaking and human capacity to adapt to environmental change. We have found that our knowledge of the physical causes and human impacts of Dust Bowl era droughts remains incomplete and that the Dirty Thirties still have much to teach us about life in the present era of global warming.

## What were “the Dust Bowl” and the “Dirty Thirties”?

**Fig. 1:**
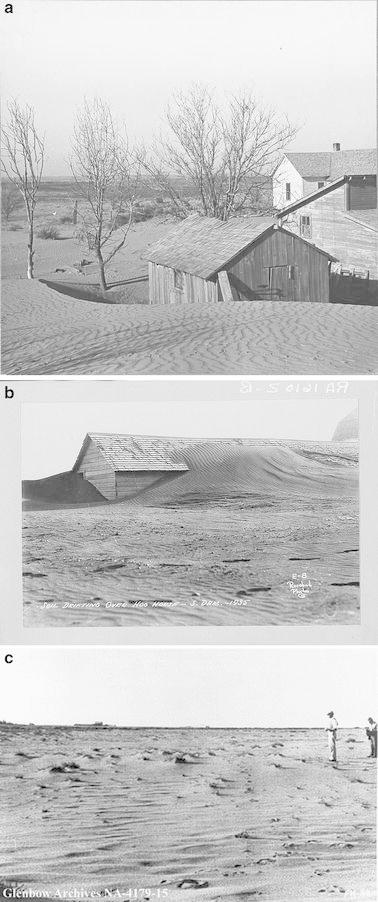
The Great Plains and the Dust Bowl proper. Great Plains boundaries based on those used by Lavin et al (2011). Outline of the Dust Bowl region is based on USDA National Resource Conservation Service wind erosion maps for 1935, 1936, and 1938, viewable at http://www.nrcs.usda.gov/Internet/FSE_MEDIA/stelprdb1049472.png

**Fig. 2:**
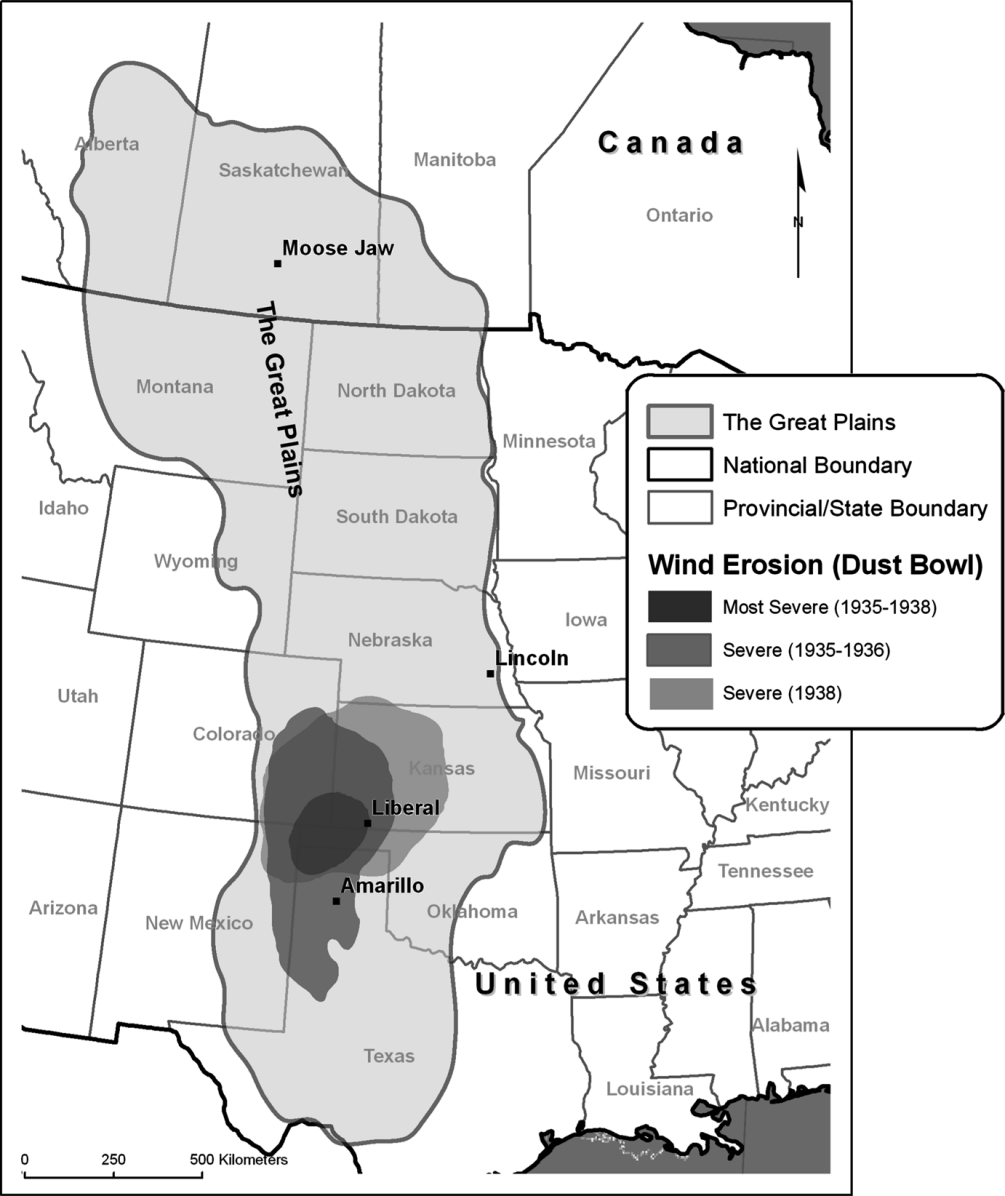
Soil blown by “Dust Bowl” winds piled up in large drifts near Liberal, Kansas. Farm Security Administration/Office of War Information Black-and-White Negatives, catalog no. LC-USF34-002504-E (b&w film nitrate neg.) (http://www.loc.gov/rr/print/res/071_fsab.html). **b** Soil drifting over hog house. South Dakota, 1935. Farm Security Administration/Office of War Information Black-and-White Negatives, catalog no. LC-USF344-001610-ZB (b&w film nitrate neg.). (http://www.loc.gov/rr/print/res/071_fsab.html). **c** Badly drifted field, Hanna area, Alberta, ca. 1930s. Glenbow Museum Archives, catalog no. NA-4179-15 (http://ww2.glenbow.org/search/archivesPhotosResults.aspx?AC=GET_RECORD&XC=/search/archivesPhotosResults.aspx&BU=&TN=IMAGEBAN&SN=AUTO25702&SE=79&RN=7&MR=10&TR=0&TX=1000&ES=0&CS=0&XP=&RF=WebResults&EF=&DF=WebResultsDetails&RL=0&EL=0&DL=0&NP=255&ID=&MF=WPEngMsg.ini&MQ=&TI=0&DT=&ST=0&IR=69393&NR=0&NB=1&SV=0&BG=&FG=&QS=ArchivesPhotosSearch&OEX=ISO-8859-1&OEH=ISO-8859-1)

The phrase “Dust Bowl” originated in a 1935 newspaper account of a tremendous dust storm that drifted across Colorado, Kansas, Oklahoma, and Texas, and was quickly adopted more widely as a term to describe that part of the southern Plains where dust storms and soil erosion were especially common and severe (Hurt 1981). The exact boundaries of the Dust Bowl are subjective. A study by Porter and Finchum (2009) found twenty-eight different published cartographical representations of the Dust Bowl, with people who actually lived on the southern Great Plains during the 1930s tending to identify its location in much the same way as did Worster (1979) in his well-known environmental history of the region, which was in turn based on US Soil Conservation Service wind erosion maps (Fig. 1). In fact, similar environmental conditions prevailed across large parts of the Great Plains that were not popularly associated with the Dust Bowl, including the Dakotas and southern portions of Alberta and Saskatchewan, Canada (Fig. 2a–c). With the passage of time, “Dust Bowl” has become more broadly and generically used to describe droughts in western North America; for example, the 2012 drought in the midwestern US spawned articles in a range of popular journals including *Forbes*, the *Herald Tribune*, *National Geographic*, the *New York Times*, and *Time* asking if a “new Dust Bowl” was upon us. Given its immediate familiarity, we use throughout the remainder of this article the phrase “Dust Bowl era” as a shorthand label for the period, although we might also have used “Dirty Thirties,” which was (and is) another widely used vernacular term describing the Great Plains during the Depression.

Referred to in Canada as “the Prairies,” the Great Plains are an extensive semi-arid ecoregion stretching from southern Texas to central Alberta in the north, covering all or part of ten US states and three Canadian provinces (Fig. 1). With a highly variable continental climate characterized by cold winters and hot, dry summers, this ecoregion was dominated by short- and mixed-grass prairie vegetation prior to European settlement (Webb 1931; Weaver 1968). Between the US Civil War and the start of the 1930s, approximately 30 % of the US portion of the Great Plains was converted to cropland, with much of the remaining grassland used for livestock grazing (Cunfer 2005). Agricultural settlement developed a few decades later on the Canadian Prairies than in the US, but similar land use patterns had emerged there as well by 1930 (Friesen 1984; Rees 1988). In both countries, governments had policies that encouraged the establishment of family-operated farms on the Great Plains through a process known as homesteading (Stroup 1988; McManus 2008). Although a number of fast-growing urban centers had developed on the Plains by the 1930s, the population remained disproportionately rural, with local livelihoods and regional economic systems tied strongly to agriculture (Hurt 2011; Waiser 2005).

From the early years of European settlement to the present day, the Great Plains have experienced episodes of drought, dust storms, downturns in the agricultural economy, and movements of people in and out of the region (Malin 1946a; Friesen 1984; Hurt 1981; 2011; McManus 2008). What made the 1930s notorious was the virtually simultaneous occurrence of harsh climatic conditions across a wide spatial area and difficult economic conditions that persisted through much of the decade. Multiple years of below average precipitation (see supplemental materials, Figures SM1 a–d), exacerbated by land management practices of the day, led to high rates of eolian soil erosion and dust storm activity across much of the region (Maio et al. 2007; Wheaton and Chakravarti 1990). The impacts of the Great Depression were experienced by Great Plains residents most directly in the forms of collapsed commodity prices, that wiped out farm incomes, and high unemployment in other economic sectors such as railroads and energy development that made non-agricultural employment opportunities scarce. The cumulative effects of the combined environmental and economic crises created widespread hardship, bankrupted many local governments, propelled high rates of farm abandonment and out-migration, and stimulated dramatic changes in government agricultural, land management and socio-economic policies in the US and Canada. Many of these are discussed in the review that now follows.

## Methods

This project began with a simple question—what have we as scholars learned in looking back on the Dust Bowl era? It was stimulated by news of the impending Ken Burns documentary film, an event that past experience has shown inevitably spurs increased popular interest in its subject (Harlan 2003), and which indeed occurred once again (Sefton 2012). We employed an established methodology for systematic literature reviews in global environmental change research (e.g., Berrang-Ford et al. 2011; Ford 2012; McLeman 2011). First, we created a questionnaire listing more specific questions for the scholarly literature, ranging from the sorts of spatial and temporal scales within which Dust Bowl research is situated, to whether any specific land-management recommendations have been made on the basis of the Dust Bowl era experience (see Supplemental Materials Q1). Some of these questions were designed to allow for simple quantitative analyses, while others could derive more qualitative details. Our next step was to create an inventory of post-1930s peer-reviewed scholarly journal articles that make explicit reference to either the “Dust Bowl” or the “Dirty Thirties” and draw directly upon events of that period as part of the reported research. Both terms (particularly “Dust Bowl”) enjoy broad usage in scholarly literature about Depression-era droughts on the Great Plains, and as keyword search terms proved to be very useful in generating the inventory.

The ISI Web of Knowledge was used for this initial stage of the research, this database having been shown elsewhere to be highly suitable for this purpose (Jasco 2005; Berrang-Ford et al. 2011). Book reviews, non-peer-reviewed studies, and articles that upon reading were found to make only passing reference to events of the Dust Bowl era were excluded. This does not imply that the contents and findings of these sources are invalid; rather, we wished to focus on those publications that would have the greatest reliability and influence within the academy and maintain consistency with other systematic review studies in the environmental change field (e.g., Ford and Pearce 2010; Ford et al. 2011). The reference list of each article in the inventory was then reviewed to identify additional scholarly articles that met the selection criteria and were not indexed in Web of Knowledge (i.e., citation tracking; for example, smaller regional history journals are not always indexed by ISI). The process was continued until no further articles were found, creating an initial inventory of 101 articles.

The questionnaire was then applied to each article using a Microsoft Excel-based form into which standardized quantitative and qualitative data were entered. The quantitative data were aggregated and analyzed to identify general trends in Dust Bowl research (see Supplemental Materials Table SM1). Qualitative data were organized by discipline and interpreted by an author generally familiar with the theories and methods used in that discipline. Key findings from each article were recorded using semi-standardized language so as to facilitate aggregation and summary. The reference lists of articles in the inventory were then resampled to identify key scholarly books that appeared on multiple occasions in reference lists of different authors and which met the selection criteria. The questionnaire process was repeated with these documents, the requirement for citation by multiple authors being to focus on those books with broad influence on the scholarship as opposed to sources drawn upon for a single research project. Given the breadth of material covered in these, the qualitative data from the questionnaire were not recorded in Excel but in separate word processor files. Finally, the inventory was supplemented by inclusion and review of several key government reports (again selected from the reference lists of authors). A full bibliography of the inventory appears in the Supplementary Materials for this article.

Our inventory contains a disproportionate number of journal articles published in the last two decades, especially in the physical sciences (see Supplemental Materials Figure SM2). Some of this growth over time can likely be attributed to the general expansion in the number of scholars and scholarly journals being published in recent decades, particularly in fields such as environmental change, earth, and atmospheric sciences. However, we also suggest in later sections of this article that the increase has also been made possible by recent developments in datasets and methodological approaches used in atmospheric sciences and global environmental change research. The results of our review and discussion of them are organized according to scholarly field in following sections, followed by a conclusion suggesting future avenues where further scholarly reflection on the Dust Bowl may yet be beneficial.

## Results and discussion

### What atmospheric science has learned from the Dust Bowl

Historian Donald Worster once wrote, “Scientists, climatologists and ecologists in particular, may one day be able to tell the historian why droughts happen” (1986, p. 109). This quote foreshadowed the fact that the greatest expansion in scholarly interest in the Dust Bowl in recent years has come in the atmospheric sciences (28 articles). Through modeling, climatological data analysis, and paleoclimate studies, two key sets of questions are the main focus of scholars working in this and related fields: what are the causes and atmospheric dynamics of the Dust Bowl and other droughts of the recent past; and, what is the return interval, intensity, and extent of past droughts (Cook et al. 2007; Schubert et al. 2008). Stimulated in part by the need to understand possible causes and impacts of anthropogenic climate change, these studies have been made possible by increasingly sophisticated global climate models and greater availability of climate datasets, especially the “reanalysis data” (Kalnay et al. 1996). Reanalysis products combine quality-controlled meteorological data, including surface, upper-air, and satellite-derived measurements, within climate models to provide theoretically consistent quantitative descriptions (i.e., three-dimensional grids) of the atmosphere, including gridded measurements interpolated to areas with or without original data (e.g., temperature) as well as derived variables such as heat flux, which are useful for understanding climate dynamics.

Atmospheric scientists have observed that droughts of comparable severity to those of the Dust Bowl era have occurred in subsequent decades, including 2011–2012, but that the 1930s droughts stand out because of their spatial extent (Karl et al. 2012). Recent studies of paleo-records have found that twentieth century droughts were shorter in duration and perhaps less severe than past Great Plains megadroughts, such as those of the sixteenth century or the tenth to thirteenth centuries AD (Cook et al. 2007; Herweijer et al. 2007). Through data analysis and modeling, the causal mechanism for Dust Bowl era droughts on the Great Plains has been linked to ocean temperature anomalies (Schubert et al. 2004; Seager et al. 2008). Specifically, it appears that Pacific sea surface temperatures (SSTs), especially as expressed by cold tropical temperatures during the La Niña phase of the El Niño Southern Oscillation (ENSO), have the most direct influence, with Atlantic Ocean SSTs perhaps having an indirect influence through dynamic effects on the atmospheric general circulation (Cook et al. 2011; Kushnir et al. 2010; McCrary and Randall 2010). Studies have identified the inherent, internal variability of the atmosphere as also having played a causal role, with local effects of dust and land surface changes having potentially intensified drought conditions during the Dust Bowl era, although the importance of these factors is still under discussion (McCrary and Randall 2010; Broennimann et al. 2009; Cook et al. 2009; Hoerling et al. 2009). The ability to predict Great Plains droughts with climate models on the basis of such information is not yet settled, with models differing in their ability to simulate droughts from a range of causes (McCrary and Randall 2010).

Instrumental and paleo-records have shown Dust Bowl era droughts to be part of a global series of precipitation anomalies (Herweijer et al. 2007). Dry conditions in western North America often coincide with dryness in mid-latitude North Atlantic regions and parts of Europe, the Middle East, and Central Asia; other regions, such as parts of the tropics, may in turn be relatively wet during such periods. Tree-ring data and lake sediment studies have also been used to study Dust Bowl era and other droughts and their effects on terrestrial and freshwater ecosystems and landscapes (Cook et al. 2007). A “drought atlas” of the past 1000 years for the US and southern Canada has been developed by assembling local-level drought reconstructions using tree-rings (Cook et al. 2007). These developments help place Dust Bowl era droughts in context, but remain as yet imperfect analogs of potential future drought conditions to be expected on the Great Plains under anthropogenic climate change (Cook et al. 2007; Herweijer et al. 2007; Seager et al. 2008). The density of paleo-records for the Great Plains region is still sparse, and there remains some debate over the likely intensity of paleo-droughts. The inherent nature of vegetation on the Plains, where trees are not abundant and are generally not long-lived, makes it challenging to generate paleo-climate chronologies using tree-ring analysis. The ability to use another key tool in paleo-climate reconstruction—lake sediment analysis—is also more challenging in the Plains than in other North American regions given the limited availability of suitable locations, the difficulty in getting reliable sub-decadal resolution, and the highly variable behavior of hydrology at local scales which in turn affects sedimentation processes (Woodhouse and Brown 2001). Despite these challenges, more paleo-research on the Great Plains is warranted and indeed necessary if atmospheric scientists are to generate better predictive tools for future regional and global drought frequency and impact.

### Looking back on the human causes of soil erosion

Over the decades, a lively debate has taken place among scholars over the human causes and contribution to the high rates of soil erosion and severe dust storms that were experienced on the Great Plains. We found eight journal articles specifically dealing with the subject, but articles in several other categories (e.g., history, multidisciplinary studies) and several books also tackle this subject. There is also a large body of government reports published by the US Department of Agriculture, the Soil Conservation Service and similar agencies, as well as detailed studies from agricultural experimental stations in Canada and the US available to scholars interested in more detailed understanding of the causes of and responses to Dust Bowl era soil erosion.

**Fig. 3:**
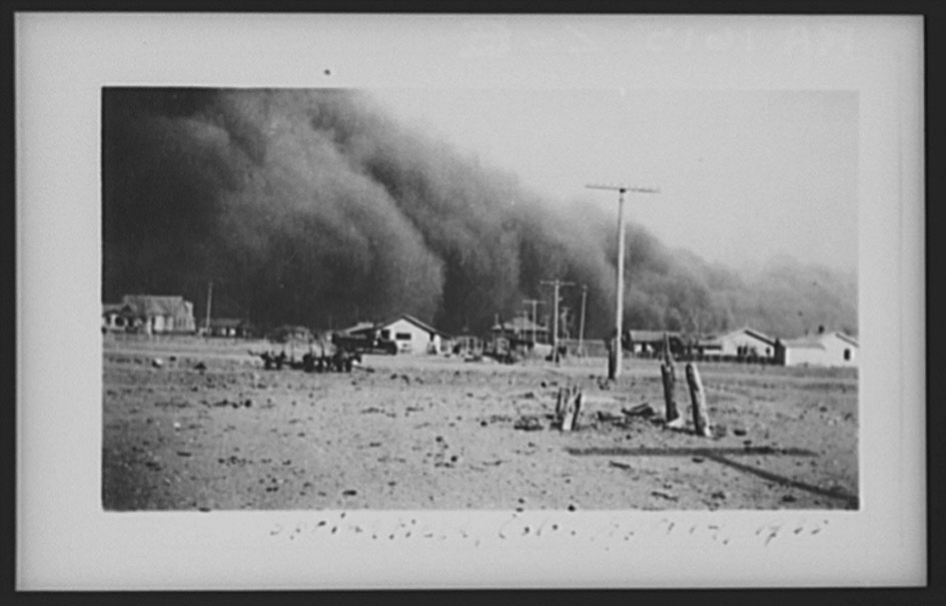
Dust storm, Baca County. Colorado Farm Security Administration/Office of War Information Black-and-White Negatives, catalog no. LC-USF34-001615-ZE (b&w film nitrate neg.) LC-USZ62-13580. (http://www.loc.gov/pictures/item/fsa1998018173/PP/)

The sources we reviewed suggest dust storms and eolian transport of soil are a natural geomorphological phenomenon on the Great Plains (Maio et al. 2007; Wheaton and Chakravarti 1990), with shallow sandy deposits being highly sensitive to variations in climate (Muhs and Holliday 1995). Soil and dust are transported by low magnitude, frequent wind events as well as less common but high magnitude storms typical of the 1930s (Lee and Tchakerian 2005; see Fig. 3). Based on written records of severe dust storms on the southern Great Plains dating back to the 1830s, before agricultural settlement took place, environmental historian James Malin (Malin 1946a, b, c) has argued that the high frequency of dust storms in the 1930s was partly a reflection of better reporting, although he did acknowledge the human contribution to the creation of dust storms through “…the initial exploitive stage of power farming, the period of the late 1920s [which] was analogous in a sense to pioneering” (1946c, p. 412). Social and natural scientists generally agree that farming practices contributed to soil erosion and dust storm occurrence, but there is a lively and ongoing debate as to the relative importance of that contribution (see, e.g., contrasting opinions among Hurt 1981; Cunfer 2005; Goudie and Middleton 1992; Worster 1979). Several scholars have suggested that supporters of New Deal agricultural policies in the US played up the role of farming practices as a cause of erosion to advance political ends (e.g., Shindo 2000, Lauck 2012) while others such as Worster (1979) place much more blame on the farming system.

A common reference point in these debates is the 1936 report of The Great Plains Committee, established by the US government to identify the causes, impacts, and necessary remedies for the crisis in the region. Table 1 summarizes the key findings—which put much of the blame on land settlement patterns and land use practices of the late nineteenth and early twentieth century—and the recommendations for action. In terms of the human contribution to the environmental disaster unfolding on the Plains, the Committee placed particular emphasis on the overgrazing of grasslands in the middle part of the nineteenth century; land speculation facilitated by government policies; the creation of land allotments under homesteading programs that were too small to be economically viable in the long term; and the failure on the part of settlers and governments alike to recognize the aridity of the climate and the diversity in soil conditions across the region. The Committee’s recommendations for action were many and placed a heavy emphasis on federal and state government intervention in land use management and soil erosion control. The Committee did not place great emphasis on irrigation or large-scale water retention projects in its recommendations, even those would turn out to be transformative adaptations in later decades, likely because the Committee did not anticipate the technological developments to come in these areas (see White 1986 for further analysis of the report).

**Table 1:** Summary of key findings of the Great Plains Committee (1936)

Outcome	Root causes	Suggested government action	Suggested farm-level action
Soil erosion	High rates of farm tenancy and absentee landlords means over-production of crops relative to livestock; soil mining; lack of farm improvement/long-term planning; expansion of farming into marginal areas; over-cultivation of small landholdings; failure to recognize diversity of soil conditions across the region	Extensive surveying of land, soil and water resources; states to create erosion control districts; create zoning regulations that direct land to appropriate use based on local conditions; expand farm extension services and agricultural research	Plow along contours; list and furrow fields at right angles to prevailing winds; plant crops in strips; terrace slopes; till soil roughly and leave high stubble after harvest; avoid bare summer fallow in wind-exposed areas and instead rotate in cover crops like clover; plant windbreaks
Loss of forage cover for grazing	Overstocking of range lands; expansion of farming into marginal areas	Federal government acquisition of range lands, with centralized control; state governments to organize grazing associations; avoid reselling rangeland seized for tax delinquency	Reduce herd sizes or keep herds off fragile lands
Inefficient use of water	Poor farming technologies and practices fail to conserve soil moisture; inadequate capacity for irrigation	Greater investment in small-scale surface water storage and retention for irrigation where possible; develop systematic irrigation policies; institute laws to protect and conserve ground water	Create deeper, better water ponds for livestock; use supplemental irrigation where cost effective to do so
Highly variable farm incomes; high rates of farm indebtedness	Undue dependence on wheat as a cash crop; high rates of tenancy; family farm landholdings too small in size; mechanization in 1920s was financed on credit during period of good rainfall and favorable crop prices	Publicly financed programs to increase farm size and active resettlement of families occupying small or marginal farms; promote development of non-agricultural resources in region (e.g., lignite coal); fund greater research into pest control	Maintain a higher of ratio of fodder and livestock to cash crops; reduce proportion of wheat and corn on farms; create diversified operational plans; keep larger feed and seed reserves

The Committee also recommended as a precursor to federal action the creation of a centralized agency to coordinate the efforts of the 25 federal agencies and many more state and local groups involved in land management on the Great Plains

With the benefit of hindsight, scholars have examined in greater detail many of the various causal factors identified in the Great Plains Committee’s report. For example, under the mantra that “rain follows the plow” (Smith 1947), late nineteenth and early twentieth century settlers plowed under large areas of native grasslands, converting these to grain, corn, and, in the southern Plains, cotton fields. The view of the Great Plains Committee was that much of this land was marginal for agriculture and should have been left as grazing range. This view, shared by some later scholars (e.g., Johnson 1947, Worster 1979), shaped many of the New Deal land management policies and programs initiated during the Depression era. However, using GIS tools not available to earlier generations of Great Plains scholars, Cunfer (2005) found that, even at the height of grassland conversion in 1935, only one-third of the US Great Plains was actually in plowed cropland, with the proportion of cropland lowest in the more arid westerns and southern portions of the Plains. The ratio of approximately 25 % cropland to 75 % grassland prevailed from the 1940s until the end of the twentieth century, a ratio that was first achieved in the 1920s (Cunfer 2005). Cunfer (2005) also found that the human contribution to eolian erosion and dust storm activity during the 1930s was most significant in the southern Texas panhandle region and that in other parts of the Great Plains conditions in the 1930s were consistent with those observed in other droughts, suggesting that Depression era land use may have been a less significant causal factor than the severity of the drought itself.

The evidence from Malin (1946c) and Cunfer (2005) supports an interpretation that the pre-Dust Bowl era was a period when farmers were learning to adjust and adapt to local conditions, with Malin (1946c) suggesting that longer-established farmers had more experience with local conditions and were better caretakers of the land than later arrivals who came during the expansion of mechanized farming across the Plains in the 1920s. Many of these latter included “suitcase farmers”—non-residents who operated monoculture grain farms as a source of speculative, often secondary income (Hewes 1973). When droughts struck, some of these ill-tended areas became sources of blowing soil that drifted across the lands of other, resident farmers (Hurt 1981). It has been observed that areas with high rates of farm tenancy often suffered from especially poor land management that contributed to soil erosion (Great Plains Committee 1936; Bonnifield 1979; Hurt 1981). While tenants and suitcase farmers clearly had less stake in the long-term health of the land, areas where most farms were operated by resident owners, such as southern Alberta and southern Saskatchewan, also suffered from erosion and dust storms (e.g., Gilbert and McLeman 2010; McLeman and Ploeger 2012). One reality is that some of the farming practices of the era used by owner-operators and non-owner-operators alike created ideal conditions for wind erosion once the droughts struck. An example of such a practice was plowing fields to a fine consistency prior to leaving them fallow, on the assumption that the exposed soil would have a higher rate of absorption and retention of moisture; instead, this practice produced conditions that made drought-desiccated soil more susceptible to wind transport (Smika 1970; Bonnifield 1979; Lyon et al. 1998). Further, the small size of Great Plains farms meant individual farmers had little influence over soil conservation in their local area and that abandoned farms became source points for erosion that adversely affected neighboring operators (Hansen and Libecap 2004).

An important question is why Great Plains farmers of the 1920s and 1930s pushed beyond the “unstable equilibrium” of cropland-to-grassland that Cunfer (2005) suggests was reached in 1920 and, with the help of irrigation in dryer areas, has been maintained from the 1940s onward. Here, the hypotheses of Donald Worster (1979, 1986) have been influential. Worster suggests that the high commodity prices triggered by World War I stimulated an entrepreneurial rush of new entrants to farming on the Great Plains and the expansion of plowed acreages by established farmers (in what was colloquially known as the “great plow-up”) (for data on wheat prices during this period, see Supplemental Materials Figure SM3). This was facilitated by developments in farm mechanization, with the credit needed to finance purchases of new equipment further drawing the Great Plains and its residents more tightly into the broader international economy. The collapse of commodity prices following 1929 stock market crash chased out the suitcase farmers, but forced remaining operators to work the land even harder to make up lost income. Simultaneously, the early 1930s saw an influx of population to rural areas, especially to those where tenant farms were available (McLeman 2006), as people displaced from other sectors of the collapsing economy looked to farming as an alternative livelihood. Thus, the arrival of drought did not so much cause the soil erosion, farm abandonments, and distress migration as reveal the socio-ecological disequilibrium that had developed on the Plains. Worster’s interpretation of the Dust Bowl is in many ways a precursor to political ecology-based interpretations of general human vulnerability to environmental change developed in subsequent years, such as Blaikie et al. (1994) pressure-and-release model and more recent “vulnerability science” approaches, which seek to identify and document the multi-scale determinants of vulnerability (Turner et al. 2003).

### Government involvement and policy intervention in land management

An important outcome of the 1930s socio-ecological crisis on the Great Plains was a greatly expanded participation of government in land management and soil conservation. In the US, a considerable range of federal agencies were involved in land management and soil conservation. The Soil Conservation Service (SCS) undertook air photo surveys and generated detailed soil maps (digitized by Cunfer (2011)) to identify areas needing attention. It sought to address the problem of wind erosion on unoccupied and abandoned lands by acquiring them outright, and running demonstration projects on terracing and contour plowing, among other activities (Johnson 1947; Hurt 1981; Bonnifield 1979; Baveye et al. 2011). Meanwhile, the US Forest Service’s Prairie States Forestry Project initiated tree-planting on private lands to create shelterbelts to reduce soil erosion, and by 1940 had planted 200 million trees on 30,000 farms from North Dakota to Texas (Johnson 1947; Gardner 2009; Hurt 1981, 1985). The Federal Emergency Relief Administration offered farmers subsidies to list-plow their lands in ways that would reduce wind erosion (Hurt 1981), while the Works Progress Administration funded infrastructure projects that included the building of dams and improved roads in rural areas (Bonnifield 1979; McLeman et al. 2008). Millions of dollars of federal investment was channeled through the US Department of Agriculture to purchase farms on what it saw to be marginally productive land (Hurt 1986; Lewis 1989; Sylvester and Rupley 2012) and in 1935 a program was initiated to create long-range weather forecasting capacity (Hecht 1983). The Resettlement Administration (later to become the Farm Security Administration) encouraged owners of small farms in dryer parts of the Plains to resettle on other lands, although participation was low because of poor financial incentives and resettlement destination lands being not much better than those left behind (Bonnifield 1979). In addition to federal efforts at changing land management practices, state governments facilitated the formation of soil conservation districts to coordinate efforts among farmers, 38 of these having been established in the southern Great Plains by 1941 (Johnson 1947).

Although the political dynamics were different in Canada, similar types of government interventions occurred in that country. For example, the Alberta government’s Special Areas Board was mandated to acquire as much farmland as possible in the dry, southeast part of the province and convert it to grazing land, and the Board today still administers 2.1 million hectares (Jones 1991; Marchildon et al. 2008). In Alberta and Saskatchewan, provincial governments subsidized the relocation expenses of families willing to abandon their farms in the drought-stricken areas (Marchildon et al. 2008; Gilbert and McLeman 2010; McLeman and Ploeger 2012). The Canadian federal government created the Prairie Farm Rehabilitation Administration in 1935 to expand government research into soil erosion and land management, carry out soil surveys, encourage farmers to adopt soil conservation measures and new farming practices, and establish shelterbelts and community pastures (Marchildon et al. 2008).

The effectiveness of these and other government programs and interventions in Great Plains land management, particularly those in the US, has been subject to debate by later scholars. Johnson’s (1947) account of government land management initiatives was generally very favorable and lamented the abandonment of many of these with the 1940s’ return of rainfall to the Great Plains and higher commodity prices stimulated by World War II. By contrast, Riney-Kehrberg (1994) and Bonnifield (1979) have observed that many US Plains farmers were suspicious of and resistant to federal land management initiatives, even in the heart of the drought. There is some evidence that soil conservation efforts initiated in the 1930s helped reduce the scale of soil erosion when drought conditions returned to the Great Plains in the 1950s, 1970s, and 1990s (Wienhold et al. 2000) and that many of the practices encouraged by government agencies are still generally appropriate for reducing dust storm activity (Ervin and Lee 1994). Bonnifield (1979) finds government programs had generally mixed results, benefitted disproportionately a relatively small number of large farm operators, and were susceptible to cronyism in many regions (the latter point also noted by McLeman et al. 2008). Hurt (1981) suggested that natural processes—the return of precipitation and the recolonization of eroded areas by plant species like Russian thistle—were likely as effective in restabilizing damaged lands as were planned interventions by the SCS. Using a reanalysis of aerial photographs, digitized soil maps and census data not available to previous scholars or to Depression-era governments, Sylvester and Rupley (2012) found that the encroachment of farms onto sub-marginal land and soils of the US Great Plains in the 1930s was relatively modest, suggesting that government efforts to acquire and reconvert farmland to grassland may have been excessive.

One outcome of Dust Bowl era government initiatives about which later scholars generally approve was the greater attention to institutional research and extension services. This has since led to the development of new erosion monitoring technologies that have been applied elsewhere (e.g., Norton and Savabi 2010) and new farming practices that emphasize protection of topsoil, such as conservation tillage, no-till farming, and the avoidance of fallowing through continuous rotational cropping (Anderson 2005; Hobbs 2007; Lal et al. 2007). Such practices minimize surface disturbance, reduce erosion, and may enable eventual remediation of lands that were damaged during the Dust Bowl era and remain so (Anderson 2005). They have been strongly recommended as a means of enhancing agricultural capacity to adapt to anthropogenic climate change in the future (Hobbs 2007), although field trials on the Great Plains show that considerable care must be taken in choosing location-appropriate crop rotations and sequences; even so, yields will continue to be variable (Lal et al 2007). Findings from Great Plains soil conservation and land management research have over the decades had influence in other parts of the world as well (Anderson 1984; Phillips 1999).

### Institutional responses to socio-ecological crisis: farm income stabilization and relief

In addition to becoming actively involved in land management, governments also became closely involved in the agricultural economy and socio-economic welfare of Great Plains residents in the 1930s. As the crisis first emerged, much of the burden of providing support to affected families fell to local governments, which quickly found they lacked the necessary resources to do so (Riney-Kehrberg 1994; McLeman et al 2008; Marchildon et al 2008). An array of social assistance, food aid, and employment-creation programs, generically referred to as “relief” by governments and residents alike, were initiated by American and Canadian governments to assist impoverished families. While not all were targeted exclusively at Great Plains communities, their impacts were particularly strong there. The US Farm Security Administration provided short-term loans to farmers to purchase food, seed, and farming supplies, overcoming the difficulty in getting credit from financial institutions (Hurt 1981). It also operated camps in California for migrant workers arriving from the Great Plains and neighboring regions (Gregory 1989). The Federal Surplus Relief Corporation supplied subsidized feed to cattle farmers, while the Resettlement Administration was providing incentives to reduce herd size (Hurt 1981). Infrastructure programs funded by the Works Progress Administration became an important source of off-farm employment in rural and urban communities across the Great Plains, while the Farm Security Administration funded the shipment and distribution of emergency food supplies to the hardest hit areas (Hurt 1981; McLeman et al 2008). As in the US, many local governments across the Canadian Prairies struggled financially during the 1930s. The provincial governments of Alberta and Saskatchewan were essentially bankrupt as well, necessitating significant federal financing of relief activities, including food assistance, rural infrastructure building, and other spending reminiscent of that which was going on in the US (Marchildon et al 2008). Scholarship since the 1930s generally agrees that such activities lessened the degree of hardship experienced by rural households across the region, although several studies (e.g., Bonnifield 1979; Gilbert and McLeman 2010; McLeman et al. 2008) emphasize the equal, if not greater, importance of household-level resilience and non-institutional social networks in successful adaptation (see “The Dust Bowl as a research analog for understanding climate adaptation and climate-related migration” subsection below).

An especially important government response to the crisis was intervention in commodity markets and production systems. In the US, a key piece of legislation was the agricultural adjustment act (AAA), which in its various incarnations created a production management system designed to stabilize commodity prices for producers, with a specific goal of restoring farm purchasing power to parity with the non-farm population by using the much higher average commodity prices of 1909–1914 as a baseline (Bowers et al. 1984). As part of this program, government provided financial incentives to farmers to withdraw less-productive lands from farming and reduce overall production to levels that provided price stability for farmers (Skopcol and Finegold 1982; Bonnifield 1979). Federally guaranteed crop insurance programs were established, with the caveat that participants had to partake in soil conservation activities (Bowers et al. 1984). When the US Supreme Court ruled the direct payments to farmers to reduce acreages to be unconstitutional, the AAA was modified and began paying farmers to increase planting of cover crops for soil conservation purposes, thereby achieving similar ends (Hurt 1981). Administration of the AAA was carried out by a special agency created within the existing US Department of Agriculture, facilitating cooperation with the Bureau of Agricultural Economics, farm extension, and the land-grant colleges. Skopcol and Finegold (1982) suggest that this organizational arrangement—which today would be called “mainstreaming” adaptation (e.g., Smit and Wandel 2006) into existing institutions—allowed the AAA to be much more effective and have a more long-lasting influence than other New Deal initiatives that were set up as independent operating agencies. Whatever the reason, Dust Bowl era agricultural policies with their heavy governmental role in commodity prices and production levels remained influential into the 1970s (see Libecap (1998) for an extensive review). When drought returned to the US Great Plains in the 1950s, many 1930s-era farm relief programs were renewed, although unlike the Dirty Thirties, the “Filthy Fifties” were not accompanied by economic recession or depressed commodity prices (Opie 1993). The 1950s also marked the beginning of widespread adoption of groundwater irrigation in many parts of the Plains, improving to some degree farmers’ ability to cope with drought.

In Canada, an important federal government intervention was the 1935 creation of a centralized marketing board for Prairie wheat and barley producers to compete with private firms for sale and distribution of grain. Membership in the new Canadian Wheat Board was initially voluntary, but during World War II made mandatory so as to strengthen government control over output and prices (Skogstad 2005). It was not until 2012 that the *de facto* monopoly of the Canadian Wheat Board over Prairie-produced wheat and barley was terminated.

### The Dust Bowl as a research analog for understanding climate adaptation and climate-related migration

In addition to documenting and analyzing the extensive intervention of government in the agricultural economy and in providing basic relief, a range of scholars have drawn attention to the initiatives and expertise of local communities and households in adapting to the conditions of the 1930s. Environmental historians were among the first to do so, through a flurry of publications released in the 1970s and early 1980s. For example, Bonnifield (1979), writing before the terms “vulnerability” and “adaptation” came into common scholarly usage, devotes a full chapter, plus many examples elsewhere, to describing how households and communities “liv(ed) through it all.” Hurt (1981) and Worster (1979) similarly wrote of challenges faced during daily life during the Dust Bowl, and how people overcame these. It is probably not coincidental that when the 1970s saw a return of drought conditions to the Great Plains, Lockeretz (1978) asked explicitly in *American Scientist* if any lessons had been learned from the Dust Bowl.

With the “critical turn” of the 1980s, scholars in natural hazards and related fields began combining political economy and other social theory with physical science methods to develop explanations of human vulnerability and adaptation to changes in the natural environment (e.g., Hewitt 1983; Blaikie 1985; see Adger (2006) for a more detailed review of the origins of vulnerability research). The Dust Bowl soon proved to be an especially useful historical analog for understanding the physical impacts and societal responses to climate change. Glantz (1988, 1991) was among the first to propose the use of the research-by-analog method for climate change impacts research and to study the Dust Bowl specifically in this fashion. Glantz (1988, along with other authors in the same edited volume) was particularly interested in how Great Plains communities would adapt to a declining availability of groundwater for irrigation—a key adaptation for many farmers on the southern Plains—with his concerns subsequently pursued by others (e.g., Opie 1992, 1993; Orlove 2005; Rosenberg et al. 1999). Rosenzweig and Hillel (1993) asked whether the Dust Bowl was an analog of the physical changes to be experienced on the Great Plains in the future and concluded that it was, except that future drought conditions would likely be worse, thereby anticipating findings generated in the subsequent bloom in Dust Bowl research by atmospheric scientists already discussed in the “What atmospheric science has learned from the Dust Bowl” section above.

The Dust Bowl era has continued to be used as a research analog in more recent years, including as a means of understanding how climate affects human migration behavior. The Dust Bowl era saw the end of decades of rural population increase on the Great Plains and initiated a trend of rural population decline that persists to this day (Parton et al. 2007). The American states of Kansas, Nebraska, North Dakota, Oklahoma, and South Dakota and the Canadian province of Saskatchewan all experienced net population losses in the 1930s (University of Virginia Geospatial and Statistical Data Center 1998; Dominion Bureau of Statistics 1936). The movement of over 300,000 people to California from Oklahoma and surrounding drought-stricken states, made famous by Steinbeck’s writings and documented by Dorothea Lange and other Farm Security Administration photographers, acquired the popular name “the Dust Bowl migration” (Gregory 1989; Lange and Taylor 1939; Stein 1973). It has since been well documented that most California-bound migrants actually originated in more densely populated (though often equally drought-stricken) areas on the eastern periphery of the Dust Bowl proper (Bonnifield 1979; Gregory 1989), although Dust Bowl counties had outmigration rates up to 15 % higher than other areas (Fishback et al. 2006; see Riney-Kehrberg (1994) for a detailed account of adaptation and migration in southwestern Kansas). And, while the California-bound migrants are the best known, tens of thousands of households migrated from rural and urban homes on the Great Plains for Washington State, Oregon, and British Columbia, and for the Aspen parklands of central Alberta and Saskatchewan (Hoffman 1938; McLeman et al 2010). A variety of internal migration patterns within the Great Plains also emerged during the 1930s, including rural-to-urban, urban-to-rural, and rural–rural migration involving tens of thousands of households, each reflecting different environmental, socio-economic and institutional dynamics operating at sub-regional and local scales (Gregory 1989; Fishback et al 2006; McLeman 2006).

Although some scholars in the 1980s suggested southern Great Plains migration developed from a unique set of dynamics (e.g., McDean 1986) or downplayed the role of environment (Manes 1982), more recently, scholars using new theories, datasets, and analytical tools have learned much in looking back upon Great Plains migration patterns of the 1930s. Gutmann et al. (2005; Gutmann and Field 2010; Deane and Gutmann 2003) have used Dust Bowl era population movements in developing explanations of the relationship between environment and American population trends more broadly, in giving context to the population displacements and migration that followed in the wake of 2005’s Hurricane Katrina, and as an inspiration for studying the relationship between dust storms and population change in later decades on the Great Plains. Fishback et al. (2006) combined newly available economic datasets with census data to assess the effects of New Deal policies on migration, finding that areas of the US where larger amounts of money were spent on public works projects, relief, and agricultural assistance, were less likely to lose out-migrants and more likely to attract migrants from elsewhere. These findings echo qualitative evidence found by McLeman et al. (2008) in eastern Oklahoma, which suggested that out-migration rates there would have been much higher if not for government assistance. McLeman and Smit (2006) used evidence from Depression-era Oklahoma to explain how migration is a means by which households adapt to climatic variability and change more generally, the likelihood of migration as opposed to other possible adaptations being subject to the influences of household access to economic, social and cultural capital. Similar findings were made in subsequent studies of Depression-era drought migrants in Alberta and Saskatchewan (Gilbert and McLeman 2010; Laforge and McLeman (2013, in press). McLeman et al (2010) and McLeman and Ploeger (2012) used GIS models that combined historical climate models, land quality inventories, and census data to identify rural areas on the Canadian Prairies where drought conditions and soil quality had a strong influence on out-migration during the 1930s, techniques that, with modification, might be undertaken elsewhere. Reuveny (2008) used the less-than-welcoming reception Dust Bowl migrants received in California (see Gregory 1989 for greater details) as one of a number of case studies to understand factors that lead environmental migrants to come into conflict with populations in receiving areas.

### Future research opportunities

Despite the large body of scholarly literature that exists, the Dust Bowl era still has much to teach us about preparing for and responding to the acute socio-environmental challenges that will continue to arise in our present era of anthropogenic climate change, food and water scarcity, and global economic uncertainty. The recent surge in interest in the Dust Bowl among climate scientists shows how more is yet to be learned about the formation, frequency, and severity of Dust Bowl-type droughts by taking advantage of newly available datasets, models, analytical tools, and computing power. Several studies described above have shown that GIS software can use digitized historical datasets to illuminate more precisely the outcomes of the complex interplay between human systems and environment on the Great Plains and suggest ways by which similar tools and data might be used for anticipating future outcomes elsewhere. Researchers have only begun to plumb the Dust Bowl experience to better understand human and institutional adaptation processes in the face of coincident environmental and economic crisis. We have also yet to explore systematically the vast wealth of community histories, autobiographical accounts, community newspaper archives, personal correspondence, and other records kept by residents of the Great Plains, which describe the innovative ways by which people adapted to “the worst hard time” (Egan 2006). It is important that scholars continue to analyze and assess such information not only because of the pace and scale of environmental change to which we must adapt in the future, but also because of the reality that cash-strapped governments have ever-less wherewithal to provide the institutional responses we have come to expect of them in the post-Dust Bowl era.

**Fig. 4:**
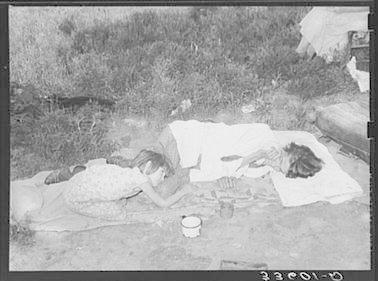
Starving woman and child in 1930s Oklahoma. The photographer’s note accompanying this image reads: “Tubercular wife and daughter of agricultural day laborer. She had lost six of her eight children and the remaining two were pitifully thin. The mother said that she had tuberculosis because she had always gone back to the fields to work within 2 or 3 days after her children were born. Shack home is on Poteau Creek near Spiro, Oklahoma.” Image source: Library of Congress, Prints and Photographs Division, FSA-OWI Collection, LC-USF34-033601-D DLC (b&w film neg.) http://www.loc.gov/pictures/collection/fsa/item/fsa2000014830/PP/

In conducting this study, we were able to answer the question “what have we learned (so far) from the Dust Bowl,” but we also noticed a decline in two particular aspects of scholarly reporting that we believe should be reversed: research studies that consider human and physical system processes together (as opposed to focussing on one or the other); and, the discussion of broader policy and planning recommendations in research findings. Although those who have first-hand knowledge of the Dust Bowl are ever more elderly and fewer in number, policy makers and the general public are familiar with it through popular culture and iconic imagery. This provides an excellent opportunity for scientists to connect their research to public dialogue about environmental change issues. Doing so, however, requires scholars already working on the Dust Bowl to make explicit the implications of their findings for policy, and requires new scholars already specialized in connecting physical and human systems research to turn their attention to the Dust Bowl. One avenue notably underrepresented in Dust Bowl scholarship to date is that of food and water security, one that is of growing global public policy concern. Here again, the Dust Bowl is recent enough to provide a powerful learning analog. While it is widely known that people can go hungry even in times and places when food is plentiful, we tend to associate that knowledge with the world’s least developed regions. It has been largely forgotten that some Americans starved during the Dust Bowl years (Fig. 4; see also McWilliams 1942; McLeman et al 2008), and it took Hurricane Katrina to remind us that food and water security issues are not restricted to the poorest parts of the planet. We could learn much about avoiding such crises in the future through further investigation of our past.

## Electronic supplementary material

Below is the link to the electronic supplementary material.

Supplementary material 1 (PDF 51 kb)

Supplementary material 2 (DOC 60 kb)

Supplementary material 3 (DOCX 244 kb)
