# Supplementary material for: What we learned from the Dust Bowl: lessons in science, policy, and adaptation
Source: Popul Environ. 2013 Aug 28;35(4):417–40. doi: 10.1007/s11111-013-0190-z (PMC4015056; doi:10.1007/s11111-013-0190-z)
Supplement: Supplementary file 1 — Supplementary material 1 (PDF 51 kb) [file 11111_2013_190_MOESM1_ESM.pdf]

Dust Bowl inventory questionnaire

Completed by:	
Date:	

TOMBSTONE DATA	ENTER DATA IN CELLS BELOW
Lead author family name:	
Year published:	
Article title:	
Journal title:	
Journal field code (see choices at column F):	
First author affiliation:	

QUANTITATIVE MEASURES		ENTER A 1 IF YES, 0 IF NO
System of primary interest:	Physical processes only (e.g. drought)	
	Human processes only (e.g. migration)	
	Both to some substantive degree of investigation	

		ENTER 1 IF YES, 0 IF NO (ONLY ONE CELL CAN = 1)
Spatial scale of process(es) of interest:	Local/community	
	Sub-regional within Great Plains	
	Great Plains region	
	North America	
	Other region or continent(s)	
	Global	

		ENTER 1 IF YES, 0 IF NO (ONLY ONE CELL CAN = 1)
Which of following time scales is the author attempting to understand the behavior of the sytem?	Long term past (i.e >100 years past)	
	Dust bowl period only (mid 1920s-WWII)	
	Recent past including Dust Bowl	
	Recent past since Dust Bowl	
	Current/ongoing processes	
	Future behavior	

	Past + present + future	
	Other (add remark)	
	Remark:	______________________

		ENTER 1 FOR YES, 0 FOR NO<br>(ENTER AS MANY 1s AS APPLY)
If a physical system/process is subject of study, which of the following is being studied	Atmospheric/climate processes	
	Biology other than agriculture	
	Crop science, other agric science excluding soil	
	Human health	
	Hydrological processes	
	Soil science, other geology/geomorphology	
	Other (add remark)	
	Remark:	______________________

		ENTER 1 FOR YES, 0 FOR NO<br>ENTER AS MANY 1s AS APPLY
If an atmospheric/climate process is subject of study, which of following is being studied (choose as many as apply)	Air circulation, patterns, current	
	Anthropogenic climate change	*
	ENSO/other climate oscillations/anomalies	
	Evapotranspiration	
	Meteorological drought	
	Modelling climate	
	Paleoclimatology	
	Other (add remark)	
	Remark:	______________________

*If you enter a 1 here, enter remark in qualitative question at line 81 below

		ENTER 1 FOR YES, 0 FOR NO<br>ENTER AS MANY 1s AS APPLY
If a human system/process is subject of study, which of following is being studied (choose as many as apply)	Agricultural processes	
	Climate adaptation	*
	Culture (includes literature)	
	Economic processes, micro	

Economic processes, macro
Gender issues
Institutional behavior (incl. law, politics)
Social behavior/social networks
Other (add remark)

Remark: ____________________

*If you enter a 1 here, enter remark in qualitative question at line 81 below

ENTER 1 FOR YES, 0 FOR NO

Does the article/author make explicit recommendations for land use/management/tenure policies or practices? (If yes, enter a note in the qualitative field at line 85)

QUALITATIVE ANALYSIS

1. What are the key conclusions of the article? Enter data in the form of "highlights" (see instruction at row 97 below)

Highlight 1:
Highlight 2:
Highlight 3:
Highlight 4 (if necessary):
Highlight 5 (if necessary):

2. In addition to the highlights entered in qualitative question 1, are there additional insights into understanding anthropogenic climate change contained in this article? If yes, add information in this field (separate ideas with semi-colons):

3. If the article makes specific recommendations for land use, management or tenure, add information in this field:

4. To facilitate writing the discussion sections of our article, enter here any additional relevant information that needs to be captured. For example, does the article riase issues that seem to have been forgotten by present day natural/social scientists? Are there any "surprise"/unexpected findings in this article? Etc
Enter point-form notes in this field:

Use this field for any additional information you wish to record for easy recall later:

INSTRUCTIONS FOR HIGHLIGHTS

Highlights consist of a short collection of 3-5 bullet points that convey the core findings of the article (maximum 100 characters per bullet point). Strive for simple terms, minimize use of adjectives, adverbs. Avoid synonyms/using multiple words to describe a common idea. Coding is simplified when the same terms are used repeatedly across bullets and across questionnaires.

Examples of highlights:

Highlight 1:	Farm abandonment during drought is higher on sandy soils
Highlight 2:	Farmers seek off-farm employment to offset lost income due to crop failure
Highlight 3:	Most who left farms migrated to cities within region
Highlight 4:	Social networks facilitate migration

Journal code	Journal field
1	Agricultural science/agronomy/crop science/vet science
2	Atmospheric science/meteorology
3	Biology/ecology/entomology (excluding agricultural sciences)
4	Demography/migration/population studies
5	Economics
6	Geography, physical: includes soil science, geomorphology, geology
7	History
8	Humanities (includes education, folklore, lierature)
9	Multidisciplinary (e.g. *Climatic Change, Global Environmental Change*
10	Political science, law, public administration
11	Water resources, water management, hydrology, irrigation
0	Other (add remark)

VOCABULARY FOR QUALITATIVE REMARKS

Cross-cutting terms	*Use instead of*
adaptation	survival, adjustment, coping, etc
agriculture	farming, crop production, etc
family	
health	illness, wellness, pneumonia
institutions	government, other formal instiutions
land tenure	sharecropping, land renting, lando pwnership etc
migration	refugee, immigration, displacement, etc
perception	subjecvtive or emotional interpretations of phenomena
poverty	destitution, economic difficulty, etc
technology	any equipment, machinery, innovations
social network	social connections, friends & relatives, etc
soil	
women	gender, farm women, etc
