# Supplementary material for: What we learned from the Dust Bowl: lessons in science, policy, and adaptation
Source: Popul Environ. 2013 Aug 28;35(4):417–40. doi: 10.1007/s11111-013-0190-z (PMC4015056; doi:10.1007/s11111-013-0190-z)
Supplement: Supplementary file 2 — Supplementary material 2 (DOC 60 kb) [file 11111_2013_190_MOESM2_ESM.doc]

**Supplementary materials – A dust bowl bibliography, assembled by McLeman et al.**

**Current as of 24 July 2013**

Anderson D (1984) Depression, Dust Bowl, Demography, and Drought: The Colonial State and Soil Conservation in East Africa during the 1930s. African Affairs 83:321–343.

Anderson RL (2005) Improving sustainability of cropping systems in the Central Great Plains. Journal of Sustainable Agriculture 26:97–114.

Archibald ES (1938) Prairie Farm Rehabilitation. CSTA Review 16:359–370.

Baveye PC, Rangel D, Jacobson AR, Laba M, Darnault C, Otten W, Radulovich R, Camargo FAO (2011) From Dust Bowl to Dust Bowl: Soils are Still Very Much a Frontier of Science. Soil Science Society of America Journal 75:2037–2048.

Biondi F, Kozubowski T, Panorska A (2005) A new model for quantifying climate episodes . International Journal of Climatology 25:1253–1264.

Blaikie P, Brookfield H (1987) Land degradation and society. Methuen, London.

Blouet BW, Luebke FC (Eds) (1979) The Great Plains: Environment and culture.

Bonnifield P (1979) The Dust Bowl: Men, Dirt and Depression. University of New Mexico Press, Albequerque.

Borchert JR (1971) The Dust Bowl in the 1970s. Annals of the Association of American Geographers 61:1–22.

Bowers, D.E., Rasmussen, W.D., Baker, G.L. (1984) History of Agricultural Price-Support and Adjustment Programs, 1933-84. US Department of Agriculture, Washington DC.

Broennimann S, Stickler A, Griesser T, Ewen T, Grant AN, Fischer AM, Schraner M, Peter T, Rozanov E, Ross T (2009) Exceptional atmospheric circulation during the “Dust Bowl”. Geophysical Research Letters

Burnette DJ, Stahle DW, Mock CJ (2010) Daily-Mean Temperature Reconstructed for Kansas from Early Instrumental and Modern Observations. Journal of Climate 23:1308.

Capotondi A, Alexander MA (2010) Relationship between Precipitation in the Great Plains of the United States and Global SSTs: Insights from the IPCC AR4 Models. Journal of Climate 23:2941–2958.

Clark JS, Grimm EC, Donovan JJ, Fritz SC, Engstrom DR, Almendinger JE (2002) Drought cycles and landscape responses to past aridity on prairies of the northern Great Plains, USA. Ecology 83:595–601.

Colvin RL (1989) Dust Bowl Legacy. Los Angeles Times Magazine 26 March 1:8–17.

Conroy JL, Overpeck JT, Cole JE, Steinitz-Kannan M (2009) Variable oceanic influences on western North American drought over the last 1200 years. Geophysical Research Letters 36:9–14.

Cook BI, Cook ER, Anchukaitis KJ, Seager R, Miller RL (2010) Forced and unforced variability of twentieth century North American droughts and pluvials. Climate Dynamics 37:1097–1110.

Cook E, Meko DM, Stahle DW, Al. E (1999) Drought Reconstructions for the Continental United States. Journal of Climate 12:1145–1162.

Cook BI, Miller RL, Seager R (2008) Dust and sea surface temperature forcing of the 1930s “Dust Bowl” drought. Geophysical Research Letters 35:1–5.

Cook BI, Miller RL, Seager R (2009) Amplification of the North American “Dust Bowl” drought through human-induced land degradation. Proceedings of the National Academy of Sciences of the United States of America 106:4997–5001.

Cook BI, Seager R, Miller RL (2010) Atmospheric circulation anomalies during two persistent North American droughts: 1932–1939 and 1948–1957. Climate Dynamics 36:2339–2355.

Cook B, Seager R, Miller RL (2011) The impact of devegetated dune fields on North American climate during the late Medieval Climate Anomaly . Geophysical Research Letters 38.

Cunfer G (2005) On the Great Plains: Agriculture and Environment. Texas A&M University Press, College Station, TX.

Cunfer G (2011) The Southern Great Plains Wind Erosion Maps of 1936-1937. Agricultural History 85:540–599.

Cutler DM, Miller G, Norton DM (2007) Evidence on early-life income and late-life health from America’s Dust Bowl era. Proceedings of the National Academy of Sciences of the United States of America 104:13244–13249.

Deane G, Gutmann MP (2003) Blowin’ down the road: Investigating bilateral causality between dust storms and population in the Great Plains. Population Research and Policy Review 22:297–331.

Debo A (1969) Prairie City: The story of an American community. Gordian Press, New York.

Dewing R (2006) Regions in Transition: The Northern Great Plains and the Pacific Northwest in the Great Depression. University Press of America, Lanham.

Easterling WE, Rosenberg NJ, McKenney MS, Jones CA (1992) An Introduction to the Methodology, the Region of Study, and a Historical Analog of Climate Change. Agricultural and Forest Meteorology 59:3–15.

Egan T (2006) The Worst Hard Time, Houghton Mifflin, New York.

Engle DM, Coppedge BR, Fuhlendorf SD (2008) From the Dust Bowl to the Green Glacier: Human Activity and Environmental Change in Great Plains Grasslands. In: Western North American Juniperus Communities. pp 253–271.

Ervin RT, Lee JA (1994) Impact of conservation practices on airborne dust in the southern High Plains of Texas. Journal of Soil and Water Conservation 49:430.

Fishback P, Horrace W, Kantor S (2006) The Impact of New Deal Expenditures on Mobility During the Great Depression . Explorations in Economic History 43:179–222.

Friesen G (1984) The Canadian Prairies: A History. University of Toronto Press, Toronto.

Fye FK, Stahle DW, Cook ER (2003) Paleoclimatic Analogs to Twentieth-Century Moisture Regimes Across the United States. Bulletin of the American Meteorological Society 84:901–909.

Fye FK, Stahle DW, Cook ER (2004) Twentieth-Century Sea Surface Temperature Patterns in the Pacific during Decadal Moisture Regimes over the United States. Earth Interactions 8:1–22.

Ganzel B (1984) Return to the Dust Bowl + Photojournal 1936-39 and 1977-79. Historical Preservation 36:32–37.

Gardner R (2009) Trees as technology: planting shelterbelts on the Great Plains. History and Technology 25:325–341.

Gilbert G, McLeman R (2010) Household access to capital and its effects on drought adaptation and migration: a case study of rural Alberta in the 1930s. Population and Environment 32:3–26.

Glantz, M.H. (ed) (1988) Societal responses to regional climatic change: Forecasting by analogy. Westview Press, Boulder CO.

Glantz MH (1990) Running on empty: Irrigation is depleting a vast reservoir under the American Great Plains. Sciences: 16–20.

Glantz, M.H. (1991) The use of analogies in forecasting ecological and societal responses to global warming. Environment 33, 10–33.

Gold JR, Revill G, Haigh MJ (1996) Interpreting the Dust Bowl: Teaching Environmental Philosophy through Film . Journal of Geography in Higher Education 20:209–221.

Goudie AS, Middleton NJ (1992) The changing frequency of dust storms through time. Climatic Change 20:197–225.

Gray JH (1967) Men against the desert. Modern Press, Saskatoon.

Great Plains Committee (1936) The future of the Great Plains. Washington DC.

Gregory JN (1989a) Dust Bowl Legacies, the Okie impact on California, 1939-1989. California History 68:74

Gregory JN (1989b) American Exodus: The Dust Bowl migration and Okie culture in California. Oxford University Press, New York.

Gregory JN (2004) The Dust Bowl Migration. In: Mink G, O’Connor A (eds) Poverty in the United States: An Encyclopedia of History, Politics, and Policy. ABC-Clio, Santa Barbara, CA,.

Gutmann MP, Deane GD, Lauster N, Peri A (2005) Two Population-Environment Regimes in the Great Plains of the United States, 1930–1990. Population and Environment 27:191–225.

Gutmann MP, Field V (2010) Katrina in historical context: environment and migration in the U.S. . Population and Environment 31:3–19.

Hale D (1982) The people of Oklahoma: Economics and social change. In: Morgan AH, Morgan HW (eds) University of Oklahoma Press, Norman, pp 31–92.

Hansen ZK, Libecap GD (2004a) Small Farms, Externalities, And The Dust Bowl Of The 1930s. Journal of Political Economy 112:665–694.

Hansen ZK, Libecap GD (2004b) Small Farms, Externalities, and the Dust Bowl of the 1930s. Journal of Political Economy 112:665–694.

Hecht AD (1983) Drought in the Great Plains: History of societal response. Journal of Climate and Applied Meteorology 22:51–56.

Henderson CA (1999) Letters from the Dust Bowl. In: Wunder JR, Kaye FW, Carstensen V (eds) University of Colorado Press, Boulder, pp 93–112.

Herweijer C, Seager R, Cook E (2007) North American droughts of the last millennium from a gridded network of tree-ring data . Journal of Climate 20:1353–1376.

Hewes L (1963) A Traverse Across Kit Carson County, Colorado, with Notes on Land-Use on the Margin of the Old Dust Bowl, 1939-1940 and 1962. Economic Geography 39:332–350.

Hewes L (1973) The suitcase farming frontier: A study in the historical geography of the central Great Plains. University of Nebraska Press, Lincoln.

Hobbs PR (2007) Conservation agriculture: what is it and why is it important for future sustainable food production? Journal of Agricultural Science 145:127–137.

Hobbs W, Fritz S, Stone J, et al. (2011) Environmental history of a closed-basin lake in the US Great Plains: Diatom response to variations in groundwater flow regimes over the last 8500 cal. yr BP . Holocene 21:1203–1216.

Hobbs P, Sayre K, Gupta R (2008) The role of conservation agriculture in sustainable agriculture . Philosophical Transactions of the Royal Society - Biological Sciences 363:543–555.

Hoehnle P (2001) Beyond the Dust Bowl: Lawrence Svobida, 1908-1984 . Agricultural History 75:271–278.

Hoerling M, Quan X-W, Eischeid J (2009) Distinct causes for two principal U.S. droughts of the 20th century. Geophysical Research Letters 36:1–6.

Hoffman, C.S. (1938) Drought and Depression migration into Oregon, 1930 to 1936. Monthly Labor Review 46, 27–35.

Hollifield M, Katon W, Spain D, Pule L (1990) Anxiety and depression in a village in Lesotho, Africa: a comparison with the United States. The British Journal of Psychiatry 156:343–350.

Hurt RD (1981) The Dust Bowl: An agricultural and social history. Nelson-Hall, Chicago.

Hurt RD (1985) The National Grasslands - Origin and Development in the Dust Bowl. Agricultural History 59:246–259.

Hurt RD (1986a) Federal land reclamation in the Dust Bowl. Great Plains Quarterly 6:94–106.

Hurt RD (2011) The Big Empty: The Great Plains in the Twentieth century. University of Arizona Press, Tuscon.

Johnson V (1947) Heaven’s Tableland: The Dust Bowl Story. Farrar, Straus and Company, New York.

Jones DC (1991) Empire of Dust. University of Alberta Press, Edmonton.

Kimmel R (1940) Unit Reorganization Program for the Southern Great Plains. Journal of Farm Economics

Knight TA, Meko DM, Baisan CH (2010) A bimillennial-length tree-ring reconstruction of precipitation for the Tavaputs Plateau, Northeastern Utah. Quaternary Research 73:107–117.

Kraenzel C (1942) New Frontiers of the Great Plains: A Cultural Approach to the Study of Man-Land Problems. Journal of Farm Economics 24:571–588.

Krim A (1992) Mother road, migrant road: Dorothea Lange on U.S. 66. Landscape 31:16–18.

Kushnir Y, Seager R, Ting M, Naik N, Nakamura J (2010) Mechanisms of Tropical Atlantic SST Influence on North American Precipitation Variability. Journal of Climate 23:5610–5628.

Laforge J, McLeman R (2013 ,in press) Social capital and drought-migrant integration in 1930s Saskatchewan. The Canadian Geographer.

Laird KR, Fritz SC, Cumming BF (1998) A diatom-based reconstruction of drought intensity , duration , and frequency from Moon Lake , North Dakota : a sub-decadal record of the last 2300 years. Journal of Paleolimnology 19:161–179.

Laird KR, Fritz SC, Maasch KA, Cumming BF (1996) Greater drought intensity and frequency before AD 1200 in the Northern Great Plains, USA. Nature 384:552–554.

Lal R, Reicosky D, Hanson J (2007) Evolution of the plow over 10,000 years and the rationale for no-till farming. Soil and Tillage Research 93:1–12.

Lange, D., Taylor, P.S. (1939) An American Exodus: A record of human erosion. Reynal & Hitchcock, New York.

Lauck J (2012). Dorothea Lange and the Limits of the Liberal Narrative: A Review Essay. Heritage of the Great Plains 45, 4–37.

Lauenroth WK, Burke IC, Gutmann MP (2000) The structure and formation of ecosystems in the North American grassland region. Great Plains Research 9:223–259.

Lavin SJ, Shelley FM, Archer JC (eds)(2011). Atlas of the Great Plains. Lincoln, University of Nebraska Press.

Lee, J.A., Tchakerian, V.P. (2005) Magnitude and Frequency of Blowing Dust on the Southern High Plains of the United States, 1947–1989. Annals of the Association of American Geographers 85, 684–693.

Lewis ME (1989) National Grasslands in the Dust Bowl. Geographical Review 79:161–171.

Libecap, G.D. (1998) The Great Depression and the Regulating State: Federal Government Regulations of Agriculture: 1884-1970. In: Bordo, M.D., Goldin, C., White, E.N. (eds) The Defining Moment: The Great Depression and the American Economy in the Twentieth Century. University of Chicago Press, Chicago, pp 181–224.

Libecap G (2007) The Assignment of Property Rights on the Western Frontier: Lessons for Contemporary Environmental and Resource Policy. The Journal of Economic History 67:257–291.

Liu G, Schwartz FW (2011) An integrated observational and model-based analysis of the hydrologic response of prairie pothole systems to variability in climate. Water Resources 47:1–15.

Lockeretz W (1978) The lessons of the Dust Bowl. American Scientist 66:560–569.

Lookingbill BD (2001) Dust Bowl, USA: Depression America and the ecological imagination, 1929-1941. Ohio University Press, Athens, OH.

Lulu M, Zobeck T, Hsieh D, et al. (2011) Optical properties of Aeolian dusts common to West Texas. Aeolian Research 3:235–242.

Lyon DJ, Stroup WW, Brown RE (1998) Crop production and soil water storage in long-term winter wheat-fallow tillage experiments. Soil and Tillage Research 49:19–27.

Maio, X., Mason, J.A., Swineheart, J.B., Loope, D.B., Hanson, P.R., Goble, R.J., Liu, X. (2007) A 10,000 year record of dune activity, dust storms, and severe drought in the central Great Plains. Geology 35, 119–122.

Malin, J.C. (1946a) Dust storms: Part one, 1850-1860. Kansas Historical Quarterly 14, 129–133.

Malin, J.C. (1946b) Dust storms: Part two, 1861-1880. Kansas Historical Quarterly 14, 265–296.

Malin, J.C. (1946c) Dust storms: Part 3, 1881-1900, concluded. Kansas Historical Quarterly 14, 391–413.

Manes S (1982) Pioneers and survivors: Oklahoma’s landless farmers. In: Morgan AH, Morgan HW (eds) Oklahoma: New Views of the Forty-Sixth State. University of Oklahoma Press, Norman, pp 93–132.

Marchildon GP, Kulshreshtha S, Wheaton E, Sauchyn D (2008) Drought and institutional adaptation in the Great Plains of Alberta and Saskatchewan, 1914–1939. Natural Hazards 45:391–411.

Masutti C (2006) Frederic Clements, climatology, and conservation in the 1930s . Historical Studies in the Physical and Biological Sciences 37:27–48.

Mauget SA (2003) Intra- to multidecadal climate variability over the continental United States:1932-99. Journal of Climate 16:2215–2231.

McCrary RR, Randall DA (2010) Great Plains Drought in Simulations of the Twentieth Century. Journal of Climate 23:2178–2196.

McDean H (1983) Social Scientists and Farm Poverty on the North American Plains, 1933-1940. Great Plains Quarterly 3:17–29.

McDean HC (1986) Dust Bowl historiography. Great Plains Quarterly 6:117–126.

McGowan DC (1975) Grassland settlers : the Swift current region during the era of the ranching frontier. Great Plains Research Centre, Regina.

McLeman R (2006) Migration Out Of 1930s Rural Eastern Oklahoma: Insights For Climate Change Research. Great Plains Quarterly 26:27–40.

McLeman R (2007) Household Access to Capital and Its Influence on Climate-Related Rural Population Change: Lessons from the Dust Bowl Years. In: Wall E, Smit B, Wandel J (eds) Farming in a Changing Climate. UBC Press, Vancouver, pp 200–216.

McLeman R, Hunter LM (2010) Migration in the Context of Vulnerability and Adaptation to Climate Change: Insights from Analogues. Wiley Interdisciplinary Reviews: Climate Change 1:450–461.

McLeman R, Ploeger SK (2012) Soil and its influence on rural drought migration: insights from Depression-era Southwestern Saskatchewan, Canada. Population and Environment 33:304–332.

McLeman R, Smit B (2006) Migration as an Adaptation to Climate Change. Climatic Change 76:31–53.

McLeman R, Herold S, Reljic Z, Sawada M, McKenney D (2010) GIS-based modeling of drought and historical population change on the Canadian Prairies. Journal of Historical Geography 36, 43–55.

McManus C (2008) History, public memory, and the land abandonment crisis of the 1920s. Prairie Forum 33:257–274.

McMillan R (1936) Some Observations on Oklahoma’s Population Movements since 1930. Rural Sociology 1:332–343.

McWilliams C (1942) Ill Fares the Land: Migrants and migratory labor in the United States. Little, Brown and Company, Boston.

Meyer WB (2000) Americans and their weather. Oxford University Press, New York.

Miner C (2006) Next Year Country: Dust to Dust in Western Kansas, 1890-1940. University Press of Kansas, Lawrence.

Mock CJ (2000) Rainfall in the garden of the United States Great Plains, 1870-1889. Climatic Change 44:173–195.

Muhs DR, Stafford TW, Swinehart JB, Cowherd SD, Mahan SA, Bush CA, Madole RF, Maat PB (1997) Late Holocene Eolian Activity in the Mineralogically Mature Nebraska Sand Hills. Quaternary Research

Narisma G, Foley J, Licker R, Ramankutty N (2007) Abrupt changes in rainfall during the twentieth century. Geophysical Research Letters 34:L06710.

Nigam S, Guan B, Ruiz-Barradas A (2011) Key role of the Atlantic Multidecadal Oscillation in 20th century drought and wet periods over the Great Plains. Geophysical Research Letters 38:1–6.

Norton BG (1990) Context and hierarchy in Aldo Leopold’s theory of environmental management. Ecological Economics 2:119–127.

Norton LD, Savabi R (2010) Evolution of a Linear Variable Intensity Rainfall Simulator for Surface Hydrology and Erosion Studies. Applied Engineering in Agriculture 26:239–245.

Opie J (1992) The drought of 1988, the global warming experiment, and its challenge to irrigation in the old Dust Bowl region. Agricultural History 66:279–306.

Opie J (1993) Ogallala: Water for a Dry Land. Lincoln, University of Nebraska Press.

Opie J (1998) Moral geography in High Plains history. Historical Geography 88:241–258.

Orlove B (2005) Human adaptation to climate change: a review of three historical cases and some general perspectives. Environmental Science Policy 8:589–600.

Parton WJ, Gutmann MP, Ojima D (2007) Long-term Trends in Population, Farm Income, and Crop Production in the Great Plains. BioScience 57:737–747.

Pegion PJ, Kumar A (2010) Multi-model estimates of atmospheric response to modes of SST variability and implication for droughts. Journal of Climate 23:4327–4341..

Philippe C. Baveye, Rangel D, Jacobson AR, Laba M, Darnault C, Otten W, Radulovich R, Camargo FAO (2011) From Dust Bowl to Dust Bowl: Soils are Still Very Much a Frontier of Science. Soil Science Society of America Journal 75:2037–2048.

Phillips ST (1999) Lessons from the Dust Bowl: Dryland Agriculture and Soil Erosion in the United States and South Africa, 1900-1950. Environmental History 4:245–266.

Plank C, Shuman B (2009) Drought-Driven Changes in Lake Areas and Their Effects on the Surface Energy Balance of Minnesota’s Lake-Dotted Landscape. Journal of Climate 22:4055–4065.

Polsky C, Easterling WE (2001) Adaptation to climate variability and change in the US Great Plains: A multi-scale analysis of Ricardian climate sensitivities. Agriculture, Ecosystems and Environment 85:133–144.

Porter JC, Finchum GA (2009) Redefining the Dust Bowl Region via Popular Perception and Geotechnology. Great Plains Research 19, 201–214.

Rees, R. (1988) New and Naked Land: Making the Prairies Home. Western Prairie Producer Books, Saskatoon.

Reuveny R (2007) Climate change-induced migration and violent conflict. Political Geography 26:656–673.

Reuveny R (2008) Ecomigration and Violent Conflict: Case Studies and Public Policy Implications. Human Ecology 36:1–13.

Rich J, Stokes S (2011) A 200,000-year record of late Quaternary Aeolian sedimentation on the Southern High Plains and nearby Pecos River Valley, USA. Aeolian Research 2:221–240.

Riebsame W (1986) The Dust Bowl Historical Image, Psychological Anchor, and Ecological Taboo. Great Plains Quarterly 1:126–136.

Riney-Kehrberg P (1989) In God we trusted, in Kansas we busted...again. Agricultural History 63:187–201.

Riney-Kehrberg P (1994) Rooted in dust: surviving drought and depression in southwestern Kansas. University Press of Kansas, Lawrence.

Riney-Kehrberg P (1992) From the Horse’s Mouth; Dust Bowl Farmers and Their Solutions to the Problem of Aridity. Agricultural History 66:137–150.

Rosenberg NJ (Ed) (1978) North American Droughts. Westview Press, Boulder.

Rosenberg, N.J., Epstein, D.J., Wang, D., Vail, L., Srinivasan, R., Arnold, J.G. (1999) Possible Impacts of Global Warming on the Hydrology of the Ogallala Aquifer Region. Climatic Change 42, 677–692.

Rosenzweig C, Hillel D (1993) The Dust Bowl of the 1930s - Analog of Greenhouse-Effect in the Great-Plains. Journal of Environmental Quality 22:9–22.

Rutledge G (2011) The “Wonder” Behind the Great-Race-Blue(s) Debate: Wright’s Eco-Criticism, Ellison's Blues, and the Dust Bowl. ANQ-A Quarterly Journal of Short Articles, Notes and Reviews 24:255–265.

Rydberg PA (1931) A short phytogeography of the Prairies and Great Plains of Central North America. Brittonia 1:57–66.

Ryves D, Battarbee R, Fritz S (2009) The dilemma of disappearing diatoms: Incorporating diatom dissolution data into palaeoenvironmental modelling and reconstruction. Quaternary Science Reviews

Sachs A (2004) Civil rights in the field: Carey McWilliams as a public-interest historian and social ecologist . Pacific Historical Review 73:215–248.

Schubert SD, Suarez MJ, Pegion PJ, Koster RD, Bacmeister JT (2004) On the cause of the 1930s Dust Bowl. Science 303:1855–1859.

Schubert, S.D., Suarez, M., Pegion, P., Koster, R., Bacmeister, J. (2008) Potential Predictability of Long-Term Drought and Pluvial Conditions in the U.S. Great Plains. Journal of Climate 21, 802–816.

Seager R, Burgman R, Kushnir Y, Clement A, Cook E, Naik N, Miller J (2008) Tropical Pacific Forcing of North American Medieval Megadroughts: Testing the Concept with an Atmosphere Model Forced by Coral-Reconstructed SSTs. Journal of Climate 21:6175–6190.

Seager R, Kushnir Y, Herweijer C, Naik N, Velez J (2005) Modeling of tropical forcing of persistent droughts and pluvials over western North America. Journal of Climate 18:4068–4091.

Seager R, Kushnir Y, Ting M, Cane M, Naik N, Miller J (2008) Would Advance Knowledge of 1930s SSTs Have Allowed Prediction of the Dust Bowl Drought? Journal of Climate 21:3261–3281.

Seager R, Naik N (2011) A mechanisms-based approach to detecting recent anthropogenic hydroclimate change. Journal of Climate 25: doi 110706140709004.

Seager R, Naik N, Baethgen W, Robertson A, Kushnir Y, Nakamura J, Jurburg S (2010) Tropical Oceanic Causes of Interannual to Multidecadal Precipitation Variability in Southeast South America over the Past Century. Journal of Climate 23:5517–5539.

Seager R, Ting M, Held I, et al. (2007) Model projections of an imminent transition to a more arid climate in southwestern North America. Science 316:1181–1184.

Seager R, Tzanova A, Nakamura J (2009) Drought in the Southeastern United States: Causes, Variability over the Last Millennium, and the Potential for Future Hydroclimate Change. Journal of Climate 22:5021–5045.

Shen S, Basist A, Howard A (2010) Structure of a digital agriculture system and agricultural risks due to climate changes. Agriculture and Agricultural Science Procedia 1:42–51.

Shindo CJ (1997) Dust Bowl migrants in the American imagination. University Press of Kansas, Lawrence.

Shindo CJ (2000) The dust bowl myth. Wilson Quarterly 24.

Skogstad, G. (2005) The Dynamics of Institutional Transformation: The Case of the Canadian Wheat Board. Canadian Journal of Political Science 38, 529–548.

Skopcol, T., Finegold, K. (1982) State Capacity and Economic Intervention in the Early New Deal. Political Science Quarterly 97, 255–278.

Smika DE (1970) Summaer Fallow for Dryland Winter Wheat in the Semiarid Great Plains. Agronomy Journal 62:15–17.

Smith HN (1947) Rain Follows the Plow: The Notion of Increased Rainfall for the Great Plains, 1844-1880. Huntington Library Quarterly 10:169–193.

Stambaugh MC, Guyette RP, McMurry ER, Cook ER, Meko DM, Lupo AR (2011) Drought duration and frequency in the U.S. Corn Belt during the last millennium (AD 992–2004). Agricultural and Forest Meteorology 151:154–162.

Stanley J (1992) Children of the Dust Bowl: The true story of the school at Weedpatch Camp. Crown Publishers, New York.

Starch EA (1939) Type of Farming Modifications Needed in the Great Plains. Journal of Farm Economics 21:114–120.

Stein WJ (1973) California and the Dust Bowl migration. Greenwood Press, Westport, CT.

Steinbeck J (1939) The Grapes of Wrath. Viking Press, New York.

Stephens PH (1937) Why the Dust Bowl? Journal of Farm Economics 19:750–757.

Stroup, R.L. (1988) Buying misery with federal land. Public Choice 57, 69–77.

Stickler A, Grant AN, Ewen T, Ross TF, Vose RS, Comeaux J, Bessemoulin P, Jylhä K, Adam WK, Jeannet P, Nagurny A, Sterin AM, Allan R, Compo GP, Griesser T, Brönnimann S (2010) The Comprehensive Historical Upper Air Network (CHUAN). Bulletin of the American Meteorological Society 91:1. doi:10.1175/2009BAMS2852.1.

Stine JK (2007) A sense of place - Donald Worster’s Dust Bowl . Technology and Culture 48:377–385.

Stock CM (1992) Main Street in Crisis: The Great Depression and the Old Middle Class on the Northern Plains. University of North Carolina Press, Chapel Hill.

Sylvester KM, Rupley ESA (2012) Revising the Dust Bowl: High Above the Kansas Grasslands. Environmental History 17:603–633.

Teisch JB (1998) From the Dust Bowl to California: the Beautiful Fraud. Midwest Quarterly - A Journal of Contemporary Thought 39:153–172.

Theobald P, Donato R (1990) Children of the Harvest: The Schooling of Dust Bowl and Mexican Migrants during the Depression Era. Peabody Journal of Education 67:29–45

Turner AO (2001) Letters from the Dust Bowl by Caroline Henderson.

Waiser, B. (2005) Saskatchewan: A New History. Fifth House, Calgary.

Warrick RA, Analysis II for AS (1980) Drought in the Great Plains: A case study of research on climate and society in the USA. In: Ausubel J, Biswas AK (eds) Pergamon Press, Oxford, pp 93–124.

Weaver, J.E. (1968) Prairie Plants and their environment. University of Nebraska Press, Lincoln.

Webb WP (1931) The Great Plains. Grosset & Dunlap, New York.

Weller KE (1995) The Dust Bowl As Place to Western Kansas Women. Social Science Journal 32:213–218.

Weller KE (1999) Historical geography of an all-black frontier town. Bulletin of the Illinois Geographical Society 41:3–25.

Wheaton, E.E., Chakravarti, A.K. (1990) Dust storms in the Canadian Prairies. International Journal of Climatology 10, 829–837.

White GF (1986) “The Future of the Great Plains” Re-visited. Great Plains Quarterly 6:84–93.

White KJC (2008) Sending or Receiving Stations? The Dual Influence of Railroads in Early 20th-Century Great Plains Settlement. Population Research and Policy Review 27:89–115. doi:10.1007/s11113-007-9056-3.

Wienhold BJ, Power JF, Doran JW (2000) Agricultural accomplishments and impending concerns. Soil Science 165:13–30.

Wilhelmi O V, Wilhite DA (2002) Assessing vulnerability to agricultural drought: A Nebraska case study. Natural Hazards 25:37–58.

Wilhite DA (1983) Government response to drought in the United States, with particular reference to the Great Plains. Journal of Climate and Applied Meteorology 22:40–50.

Wilhite DA, Wood DA (2001) Revisiting drought relief and management efforts in the West: Have we learned from the past? Journal of the West 40:18–25.

Windschuttle K (2002) Steinbeck’s myth of the Okies. The New Criterion 20. <http://www.newcriterion.com/archive/20/jun02/steinbeck.htm>.

Woodhouse C, Brown PM (2001) Tree-ring evidence for Great Plains drought. Tree-ring Research 57, 89–103.

Woodhouse C, Overpeck JT (1998) 2000 Years of Drought Variability in the Central United States. Bulletin of the American Meteorological Society 79:2693–2714.

Worster D (1979) Dust Bowl: The Southern Plains in The 1930s. Oxford University Press, New york.

Worster D (1986) The Dirty Thirties: a study in agricultural capitalism. Great Plains Quarterly 6:107–116.

Wunder JR, Kaye FW, Carstensen V (1999) Americans view their Dust Bowl experience. University of Colorado Press, Boulder.

Wyckoff PH, Bowers R (2010) Response of the prairie forest border to climate change: impacts of increasing drought may be mitigated by increasing CO2. Journal of Ecology 98:197–208.

Zeng N, Yoon J-H, Mariottie A, Al. E (2008) Variability of basin-scale terrestrial water storage from a PER water budget method: The Amazon and the Mississippi. Journal of Climate 21:248–265.

Zhang Z (2005) Coupled patterns of spatiotemporal variability in Northern Hemisphere sea level pressure and conterminous U.S. drought. Journal of Geophysical Research 110:1–12.

Zhang ZH, Mann ME, Cook ER (2004) Alternative methods of proxy-based climate field reconstruction: application to summer drought over the conterminous United States back to AD1700 from tree-ring data . Holocene 14:502–516.

Zhang B, Schwartz FW, Liu G (2009) Systematics in the size structure of prairie pothole lakes through drought and deluge. Water Resources Research 45.
