# Supplementary material for: What we learned from the Dust Bowl: lessons in science, policy, and adaptation
Source: Popul Environ. 2013 Aug 28;35(4):417–40. doi: 10.1007/s11111-013-0190-z (PMC4015056; doi:10.1007/s11111-013-0190-z)
Supplement: Supplementary file 3 — Supplementary material 3 (DOCX 244 kb) [file 11111_2013_190_MOESM3_ESM.docx]

**Supplementary materials: Additional figures and tables, McLeman et al.**

Figures SM1a-d: Precipitation data for selected Great Plains locations, 1930s.


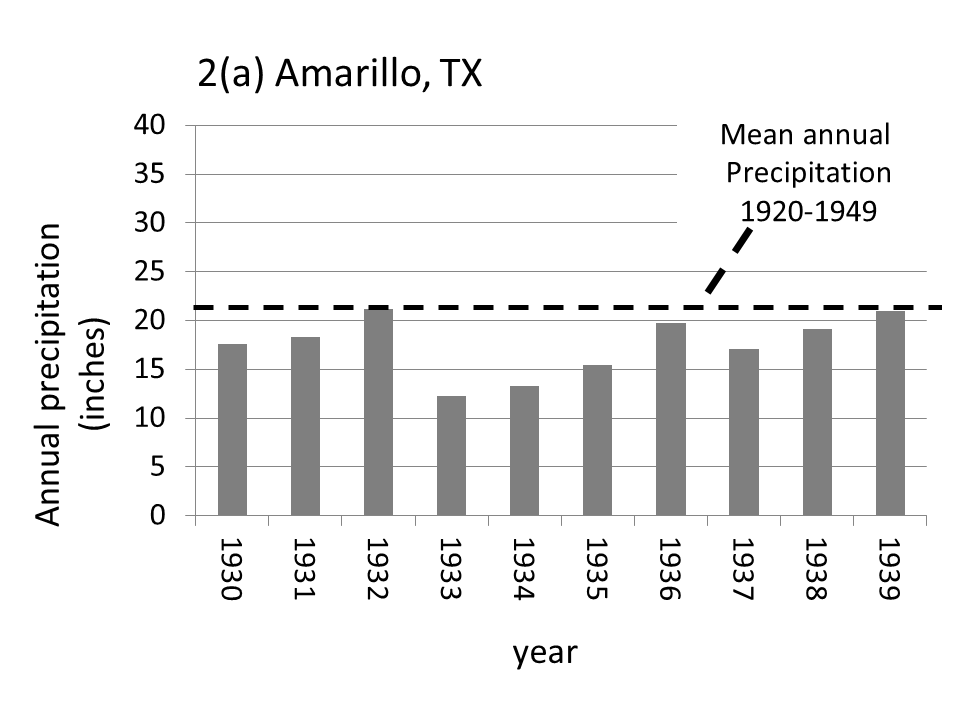


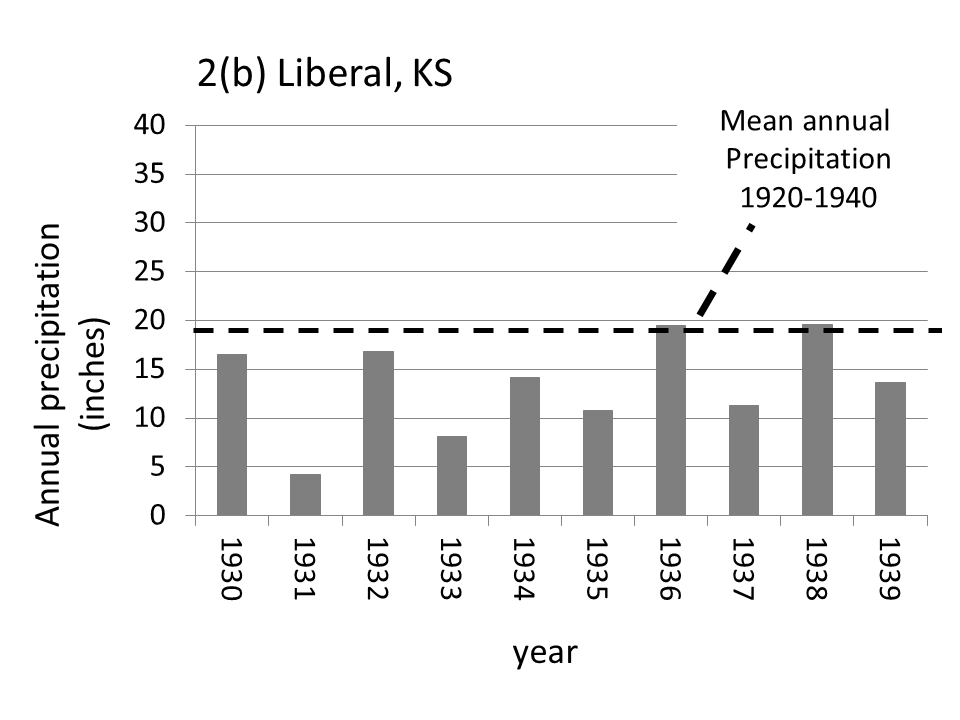


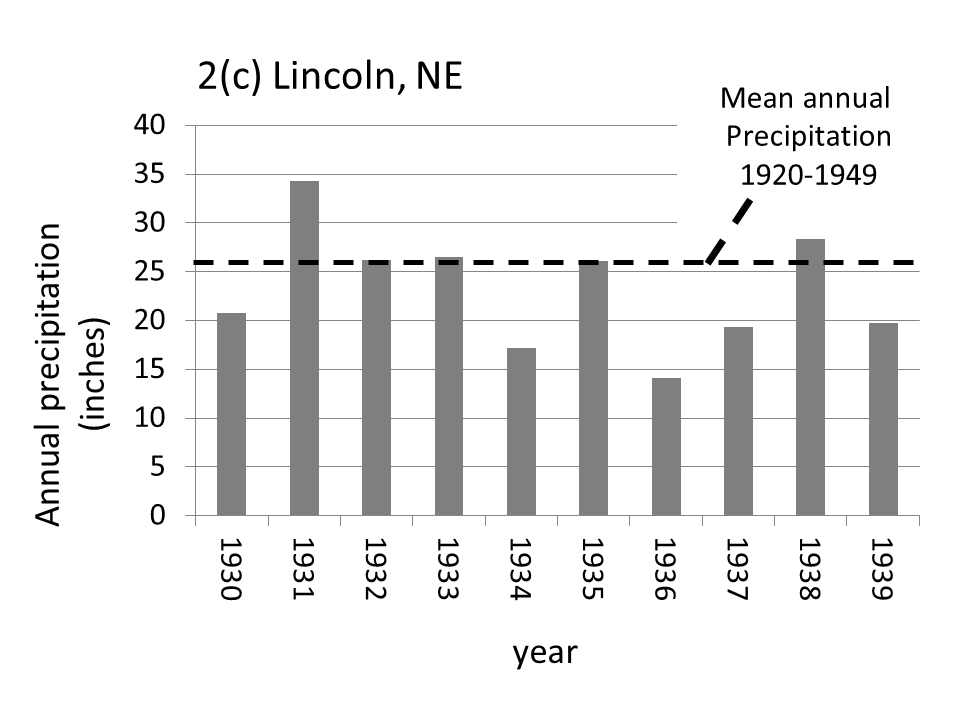


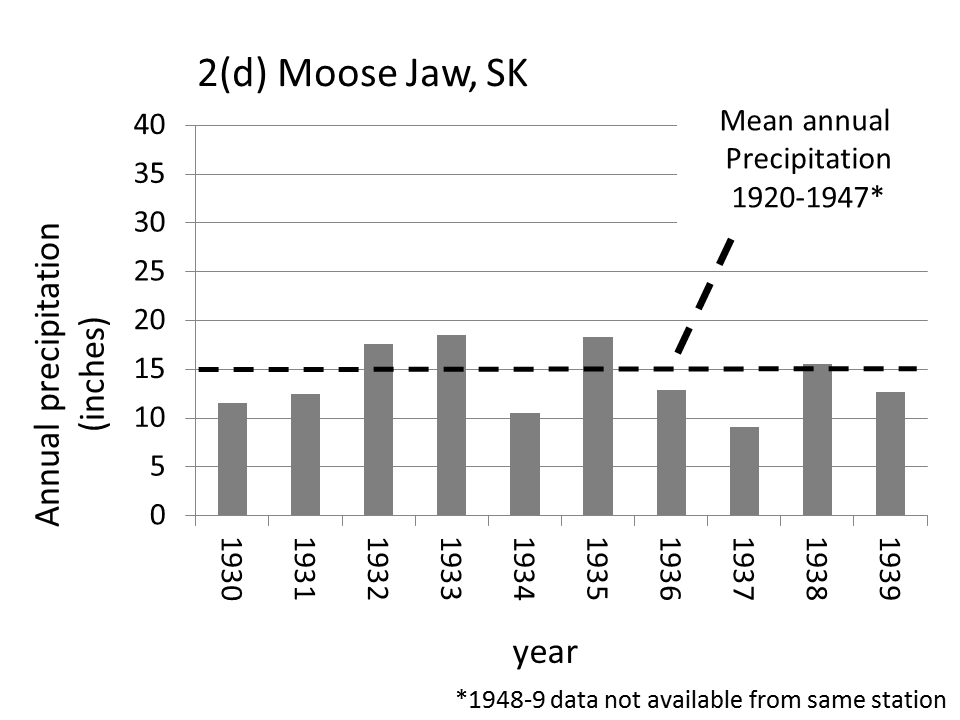


Caption: Precipitation data sources: National Weather Service Weather Forecast Office Amarillo TX (2012) *Yearly Precipitation Totals 1921-1960*, <http://www.srh.noaa.gov/ama/?n=yearly_precip>; Western Regional Climate Center (2013) *Historical Data,* <http://www.wrcc.dri.edu/>; University of Nebraska - Lincoln Weather Center (2012) *Lincoln Weather and Climate*, <http://snr.unl.edu/lincolnweather/data/monthly-precipitation.asp>; Environment Canada (2012) *National Climate Data and Information Archive*, <http://climate.weatheroffice.gc.ca/>

Table SM1: Summary statistics, scholarly journal articles in inventory

| **Variable** | **Publications^*^** |
| --- | --- |
| **Topic category** |  |
| Atmospheric science/meteorology | 28 |
| Multidisciplinary (e.g. *Climatic Change, Global Environmental Change*) | 23 |
| History | 10 |
| Agricultural science/agronomy/crop science/vet science | 8 |
| Geography, physical: includes soil science, geomorphology, geology | 8 |
| Humanities (includes education, folklore, literature) | 7 |
| Demography/migration/population studies | 5 |
| Economics | 5 |
| Biology/ecology/entomology (excluding agricultural sciences) | 4 |
| Water resources, water management, hydrology, irrigation | 2 |
| Political science, law, public administration | 1 |
|  |  |
| **System of Primary Interest** |  |
| Physical processes only (e.g. drought) | 52 |
| Human processes only (e.g. migration) | 27 |
| Both to some substantive degree of investigation | 22 |
|  |  |
| **Spatial Scale of Interest** |  |
| Global | 20 |
| Sub-regional within Great Plains | 26 |
| Great Plains region | 23 |
| North America | 16 |
| Local/community | 9 |
| Other region or continent(s) | 7 |
|  |  |
| **Which of the following time scales is the author attempting to understand the behaviour of the system?** |  |
| Recent past including Dust Bowl | 43 |
| Dust Bowl period only (mid 1920s-WWII) | 18 |
| Long term past (i.e >100 years past) | 17 |
| Current/ongoing processes | 8 |
| Past + present + future | 11 |
| Other | 6 |
|  |  |
| **If a physical system/process is subject of study, which of the following is being studied?**** |  |
| Atmospheric/climate processes | 42 |
| Soil science, other geology/geomorphology | 26 |
| Crop science, other agricultural sciences excluding soil | 11 |
| Hydrological processes | 7 |
| Other (add remark) | 4 |
| Biology other than agriculture | 2 |
|  |  |
| **If an atmospheric/climate process is subject of study, which of the following is being studied?^2^** |  |
| Meteorological drought | 29 |
| Air circulation, patterns, current | 26 |
| ENSO/other climate oscillations/anomalies | 20 |
| Modelling climate | 18 |
| Paleoclimatology | 12 |
| Evapotranspiration | 9 |
| Anthropogenic climate change | 6 |
| Other | 4 |
|  |  |
| **If a human system/process is subject of study, which of the following is being studied^2^** |  |
| Agricultural processes | 36 |
| Institutional behavior (incl. law, politics) | 32 |
| Climate adaptation | 25 |
| Culture (includes literature) | 17 |
| Economic processes, micro | 17 |
| Economic processes, macro | 11 |
| Social behavior/social networks | 11 |
| Gender issues | 4 |
| Other | 17 |
|  |  |
| **Does the article/author make explicit recommendations for land use/management/tenure policies or practices** |  |
| Yes | 13 |
| No | 88 |

^*^ *Total number of publications is provided. Given the total number of publications (101), this value approximately percentage of publications.*

^**^ *These categories are not mutually exclusive, and therefore results do not add up to the total number of publications (101). For these questions, all that apply were selected.*

Figure SM2: Number of journal articles in inventory, by publication date and scientific field of interest


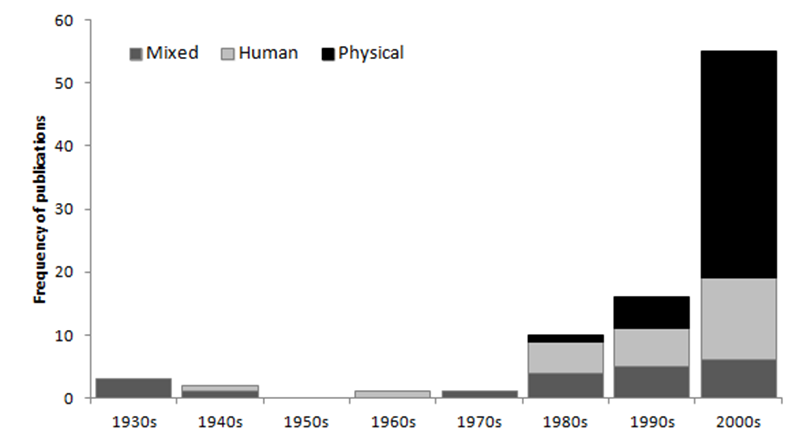


Figure SM3: Average wholesale wheat prices in Canada, 1914-1939


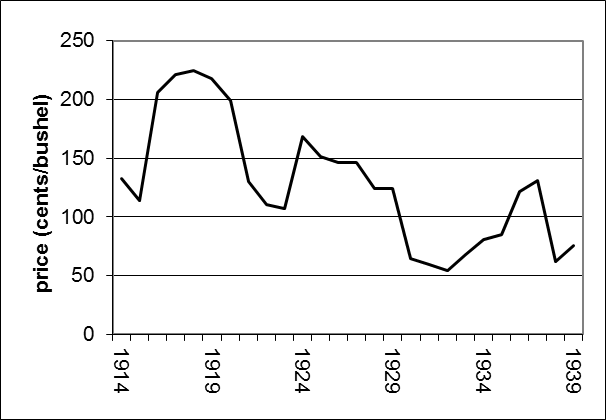


Caption: Data source: Statistics Canada, Historical Statistics of Canada, Series M228-238, Wholesale market prices for selected agricultural products, 1867 to 1974, online at http://www.statcan.ca/english/freepub/11-516-XIE/sectionm/M228_238.csv. Reproduced with permission from McLeman, R., Herold, S., Reljic, Z., Sawada, M., McKenney, D. (2010) GIS-based modeling of drought and historical population change on the Canadian Prairies. J Hist Geogr 36, 43–55.
